# Ketogenic Diet Improves Forelimb Motor Function after Spinal Cord Injury in Rodents

**DOI:** 10.1371/journal.pone.0078765

**Published:** 2013-11-04

**Authors:** Femke Streijger, Ward T. Plunet, Jae H. T. Lee, Jie Liu, Clarrie K. Lam, Soeyun Park, Brett J. Hilton, Bas L. Fransen, Keely A. J. Matheson, Peggy Assinck, Brian K. Kwon, Wolfram Tetzlaff

**Affiliations:** 1 International Collaboration on Repair Discoveries (ICORD), Blusson Spinal Cord Center, Vancouver, British Columbia, Canada; 2 Department of Zoology, University of British Columbia, Vancouver, British Columbia, Canada; 3 Combined Neurosurgical and Orthopaedic Spine Program, Department of Orthopaedics, University of British Columbia, Vancouver, British Columbia, Canada; Hertie Institute for Clinical Brain Research, University of Tuebingen., Germany

## Abstract

High fat, low carbohydrate ketogenic diets (KD) are validated non-pharmacological treatments for some forms of drug-resistant epilepsy. Ketones reduce neuronal excitation and promote neuroprotection. Here, we investigated the efficacy of KD as a treatment for acute cervical spinal cord injury (SCI) in rats. Starting 4 hours following C5 hemi-contusion injury animals were fed either a standard carbohydrate based diet or a KD formulation with lipid to carbohydrate plus protein ratio of 3:1. The forelimb functional recovery was evaluated for 14 weeks, followed by quantitative histopathology. Post-injury 3:1 KD treatment resulted in increased usage and range of motion of the affected forepaw. Furthermore, KD improved pellet retrieval with recovery of wrist and digit movements. Importantly, after returning to a standard diet after 12 weeks of KD treatment, the improved forelimb function remained stable. Histologically, the spinal cords of KD treated animals displayed smaller lesion areas and more grey matter sparing. In addition, KD treatment increased the number of glucose transporter-1 positive blood vessels in the lesion penumbra and monocarboxylate transporter-1 (MCT1) expression. Pharmacological inhibition of MCTs with 4-CIN (α-cyano-4-hydroxycinnamate) prevented the KD-induced neuroprotection after SCI, In conclusion, post-injury KD effectively promotes functional recovery and is neuroprotective after cervical SCI. These beneficial effects require the function of monocarboxylate transporters responsible for ketone uptake and link the observed neuroprotection directly to the function of ketones, which are known to exert neuroprotection by multiple mechanisms. Our data suggest that current clinical nutritional guidelines, which include relatively high carbohydrate contents, should be revisited.

## Introduction

Previously, we have shown that Every-Other-Day-Fasting (EODF) improves neurological recovery in rats following cervical and thoracic SCI [1-3]. Despite this demonstration of neuroprotective and functional benefits of EODF, concerns have been voiced regarding the additional weight loss such a dietary regime would likely induce in acute SCI patients at a time when they are already nutritionally vulnerable and prone to losing weight. Therefore, we explored the implementation of an alternative dietary strategy that mimics the increase in neuroprotective ketone bodies as that observed with EODF, albeit while consuming food.

 Ketogenic diets (KD) are high in fat while carbohydrates are kept at a minimum level. A physiologically low intake of carbohydrates causes a metabolic state of increased hepatic ketogenesis, which results in high levels of blood ketone bodies (β-hydroxybutyrate, acetoacetate and acetone) due to the breakdown of fatty acids [4,5]. These ketone bodies cross the blood brain barrier and enter neuronal and glial cells through monocarboxylic acid transporters (MCTs) of which MCT1 is the primary isoform found in astrocytes, oligodendrocytes, and endothelial cells. After uptake into the mitochondria, ketone bodies are converted to acetyl-CoA, to enter the tricarboxylic acid pathway for ATP generation [6-13]. 

KD was discovered in the 1920 for the treatment of epilepsy and only recently validated as an effective non-pharmacological preventive treatment for drug-resistant epilepsy. Interestingly, a recent pilot study suggested that KD might also be beneficial in humans with Parkinson’s disease as significant improvement on the Unified Parkinson's Disease Rating Scale were seen after as little as 28 days [14]. In the laboratory setting, KD has recently been shown to be effective in animal models of several neurodegenerative diseases including amyotrophic lateral sclerosis, Alzheimer’s disease, cerebral hypoxia or global brain ischemia, cerebral artery occlusion, and traumatic brain injury [15-19]. 

 To date, the potential benefits of KD have never been tested in the context of SCI. Hence, we assessed the effectiveness of KD comprised of a 3:1 ratio of fat to carbohydrate plus protein in which the carbohydrate content is limited to 3% by wet weight. Since most clinical injuries occur at the cervical level of the spinal cord, we tested KD in a C5 hemi-contusion model in rats. We used a wide variety of behavioral and histological assays to test the effects of KD after SCI. To understand how KD might ameliorate SCI symptoms, we additionally examined the effect of pharmacological inhibition of MCTs after SCI. We hypothesized that inhibition of MCT transport will attenuate the beneficial effects of KD supporting the notion that the benefits of this diet are directly mediated by ketone body uptake. 

## Materials and Methods

All Animal experimentation was performed in accordance with the Guidelines of The Canadian Council for Animal Care and was approved by the Animal Care Committee of the University of British Columbia (Permit Number: A010-0018). All efforts were made to minimize suffering and the number of animals used. All surgery as well as behavioural and histological analyses were performed blinded to the treatment assignment of the animals.

### Animals and general housing conditions

Upon arrival young adult male Sprague-Dawley rats (~350g; Charles River Breeding Laboratory) were group-housed (21°C; 12h:12h light:dark cycle) and given *ad* *libitum* access to standard rodent diet (LabDiet-5001; Purina Mills Brentwood, MO).

### C5 hemi-contusion

This was performed as described previously [20]. In brief, after a unilateral C5 laminectomy, the dorsal neural processes of C4-C6 were held with a custom designed clamp and rigidly fixed in a frame tilted at a 25.0° angle. The contusion force was set to 150 kdyne while reaching velocities around 100mm/s using the IH impactor [21]. In experiment 2, the injury was performed on the side of the preferred reaching paw as assessed with the Whishaw reaching test prior to injury [22]. 

### Grouping and dietary regimen after SCI

Four hours after injury, the animals were randomized into either: 1) the standard diet (SD) group, with continued *ad libitum* access to regular carbohydrate-based rodent diet (F5960; Bio-Serve, Frenchtown, NJ), or 2) the 3:1 KD group in which the animals were fed a KD diet with ratio of fat to carbohydrate+protein of 3:1 (F5848; paste; Bio-Serv; Frenchtown, NJ). For further details on diets and experimental groupings see Table 1-3. 

**Table 1:** Energy distribution of control and ketogenic diets.

	**SD**	**3:1 KD**
	F5960	F5848
**Distributor**	Bio-Serv	Bio-Serv
**% (w/w)**		
Fat	5.0	65.8
carbohydrate	65.4 ^a^	3.0 ^b^
protein	18.1	18.1
**kcal/g**		
fat	0.45	5.92
carbohydrates	2.62	0.12
protein	0.72	0.72
**total**	3.79	6.76

^a^ carbohydrates contributed to formula mineral mix AIN-76: 0.0045 kg sucrose/kg diet; vitamin mix: 0.0204 kg sucrose/kg diet; dextrose: 0.0076 kg/kg diet; total carbohydrates: 3.25% w/w

^b^ carbohydrates contributed to formula mineral mix AIN-76: 0.0079 kg sucrose/kg diet; vitamin mix: 0.0186 kg sucrose/kg diet; total carbohydrates: 2.65% w/w

**Table 2:** Fatty acid composition of control diets and ketogenic diets.

	**SD**	**3:1 KD**
	F5960	F5848
**Fatty acid profile** (% w/w)		
saturated	1.9	25.2
monounsaturated	1.8	24.0
polyunsaturated	0.77	10.2
n-3	0.04	0.56
n-6	0.73	9.6

**Table 3:** Overview of the experimental studies.

**Exp.**		**Groups**	**Species**	**Treatment duration**	**Outcome parameters**
**1**	n=10	1) 3:1KD	Sprague Dawley	0-14 wpi	Body weight, blood parameters
	n=9	2) SD			*Behavior*: cylinder rearing, grooming test
					*Histology*: lesion area, tissue sparing
**2**	n=18	1) 3:1KD > SD	Sprague Dawley	0-12 wpi *	*Behavior*: Cylinder rearing, grooming test, Montoya staircase, Whishaw reaching
	n=18	2) SD		*12-16 wpi Switched back to SD	
**3**	n=13	1) 3:1KD	Sprague Dawley	0-12 wpi	*Behavior*: Montoya staircase
	n=14	2) SD			
**4**	n=4	1) 3:1KD	Sprague Dawley	0-7 dpi	*Histology*: Immunofluorescence for MCT1 and GLUT1
	n=5	2) SD			
**5**	n=4	1) 3:1KD	Sprague Dawley	0-14 dpi	*Biochemical*: RT-PCR for BDNF, PDGFβ, VEGF, HIF1α, SDF1α
	n=4	2) SD			
**6**	n=4	1) 3:1KD	Sprague Dawley	0-14 dpi	*Biochemical*: Western Blotting for MCT1
	n=5	2) SD			
**7**	n=5	1) 3:1KD	C57Bl/6	0-7 dpi	*Histology*: Tissue sparing
	n=4	2) SD			
	n=6	3) 3:1KD+4-CIN			
	n=6	4) SD+4-CIN			

Abbreviations: BDNF, brain-derived neurotrophic factor; dpi: days post spinal cord injury; 4-CIN, Alpha-cyano-4-hydroxycinnamic acid (MCT1 inhibitor); GLUT1: glucose transporter-1; HIF1α, hypoxia-inducible factor-1α; KD: ketogenic diet; MCT1: monocarboxylate transporter isoform 1; PDGFβ, platelet-derived growth factor-β; SD: standard diet; SDF1α, stromal cell-derived factor -1α; VEGF, vascular endothelial growth factor; wpi: weeks post spinal cord injury.

### Food intake

The average caloric intake was calculated by multiplying daily food intake per cage over a 24-hour period with the calorie density of the appropriate diet divided by number of rats in the cage. 

### Blood ketone levels

To measure blood β-hydroxybutyrate concentration (mmol/L), Medisense Precision Xtra monitor (Abbott Laboratories, Canada) was used with blood obtained by tail vein puncture. 

### Cylinder rearing task

Details on forelimb usage during rearing have been described previously [23,24]. The animals were videotaped during spontaneous vertical exploration in a plexi-glass cylinder, and a frame-by-frame analysis of the forelimb usage during 20 independent rears was performed. During a rearing motion, paw usage of the affected forelimb was scored as “ipsilateral only“, or “both (ipsi+contra)” usage. 

### Grooming test

Grooming is an innate behavior and was assessed as previously described in detail [25]. After placing a drop of water onto the head of the rat, grooming activity was recorded with a video camera for a total time of 15 minutes. Frame by frame video playback was used to score each forelimb independently by the maximal range of motion during grooming. 

### Montoya staircase reaching task

The Montoya staircase is a device for the assessment of food pellet reaching [26,27]. Animals were placed in the Montoya staircase apparatus for a period of 15 minutes, after which the number of food pellets consumed on either the right or left side was noted (Dustless Precision Pellets F06506; Bio-Serv). To increase reaching motivation, caloric intake was reduced to 75% of food consumed during *ad libitum* feeding conditions. Pellets were color-coded according to the level of each well on the staircase to distinguish between pellets remained and pellets misplaced [28].

### Whishaw single pellet reaching task

The Whishaw chamber consists of a plexi-glass box with a narrow slit through which the rats can reach for a single food pellet placed onto a platform attached to the front of the box. This platform has two indentations for the food pellets that are placed in such a manner that one can only be reached with the left paw and the other only with the right paw. High fat food pellets (Dustless Precision Pellets F06506; Bio-Serv) were placed in either the left or right indentations depending on pre-operatively measured paw preference. A total of 10 separate reaching attempts were videotaped and scored for 7 distinct reaching components as described previously [29,30]. Qualitative measures were analysed frame-by-frame. 

### Immunohistochemistry

Paraformaldehyde perfusion-fixed and cryoprotected segments of the spinal cord (C3-C7) were frozen and cut longitudinally (horizontal) on a cryostat (Microm, Heidelberg, Germany) at 20μm. Slides were incubated with either GFAP (1:000; Z0334; DakoCytomation, Carpinteria, CA), endothelial cell antigen 1 (RECA-1; 1:250; MCA970R; AbD Serotec, Oxford, UK), GLUT1 (1:200; Sc-1605, Santa Cruz Biotechnology, Santa Cruz, CA) or MCT1 antibody (1:500; cat. no. AB1286; Millipore Corporation, Billerica, MA). For detection, Alexa Fluor 488-, 594-, or 405-conjugated secondary antibodies were used (Jackson ImmunoResearch Laboratories, Inc., West Grove, PA).

### Assessment of lesion size

Lesion area of longitudinal sections was based on the disruption of the cytoarchitecture using GFAP immunostaining. Total lesion volume (mm^3^) was calculated with the Cavalieri's Estimator of Morphometric Volume on 7x 20 μm thick tissue sections spaced 240 μm apart through the dorso-ventral axis of the spinal cord: [SUM lesion area * distance between sections]-[lesion area epicenter * section thickness] [31]. 

### Assessment of vascular density

Immunostaining for RECA-1 and GLUT1 were used to characterize blood vessel density [32]. Digitized photographs at the level of the spinal central canal were used to count the number of positive blood vessels within a 750×750μm window placed over the gray matter at the penumbra of the lesion (with bottom of the window always touching boundary between gray and white matter). 

### Antrograde tracing of CST axons

To anterogradely trace ipsilateral CST axons, 10% FluoroEmerald (Molecular Probes) was injected into the forelimb area of the sensory-motor cortex (4 injection sites, 0.4 μl each) as described previously [1-3]. Tracer filled ipsilateral CST axon branches in the gray matter (1 mm rostral and 2 mm caudal to the lesion edge) were digitally tracked in 7 longitudinal spinal cord sections at 80 μm intervals centered on the epicenter. Sections were quantified and the total length of CST axon branches in the gray matter at the C5 level for each animal was normalized to their C1 CST average intensity to account for differences in tracing efficacy.

### Analysis of tissue sparing

Three microscope pictures were taken for quantification, all at the epicentre, 80 μm apart within the dorsal-ventral position of the corticospinal tract (CST). Spared grey matter was measured as the minimum width of gray matter between the border of the lesion and the ipsilateral CST, i.e. the width of the spared dorsal horn (sum of all three sections). White matter sparing was defined as the minimum width of the residual ipsilateral CST (sum of all three sections). 

### Western blotting

15 μg of ipslateral spinal cord protein samples 7 days post-injury (centered on the lesion plus 2.5 mm rostral and caudal of the spinal cord penumbra) were separated by standard SDS-PAGE electrophoresis and blotted onto PDVF membranes and incubated with MCT1 (1:500; AB1286; Millipore Corporation, Billerica, MA) or actin antibody (1:2000; 69100; ICN Biomedicals Inc., Solon, OH). Protein density was calculated from digital images (BioSpectrum AC Imaging System; UVP, Upland, CA) with Sigma Scan Pro 4 Software (Chicago, IL) and presented as the MCT1/actin mean optical density ratio.

### Isolation of total RNA and real-time quantitative RT-PCR

Like for the protein analysis a 5 mm segment of the ipsilateral side of the spinal cord centered on the impact site was removed for extraction of RNA. Total RNA was extracted using a Trizol Reagent (Invitrogen, CA, USA) according to the manufacturers’ manual. Quantitative PCR reactions were performed using the TaqMan Fast Universal PCR Master Mix Kit (Applied Biosystems, CA, USA). The mRNA for Hypoxia Inducible Factor-1α (HIF1α), brain-derived neurotrophic factor (BDNF), vascular endothelial growth factor (VEGF), platelet-derived growth factor-β (PDGFβ) and stromal cell-derived factor-1α (SDF1α) was measured by real-time quantitative RT-PCR using PE Applied Biosystems prism model 7700. Triplicate reactions were run for each sample for both the gene of interest and the endogenous control (glyceraldehyde-3-phosphate dehydrogenase; GAPDH). Table 4 contains the TaqMan primer and probe sequences for the genes analyzed. Results are presented as 2^-dCt^, where dCt was difference between cycle threshold (Ct) values of GAPDH and gene of interest, a calculation that compensates for loading errors. Subtraction of dCt of SD rats from dCt of KD rats gives the ddCt value that was used to calculate relative expression levels in KD animals (2^-ddCt^). 

**Table 4:** Genes analyzed by quantitative TaqMan PCR.

**Gene**	**Primer/Probe**	**Sequence**
BDNF	Forward primer	ACCATAAGGACGCGGACTTG
	Reverse primer	GAGGCTCCAAAGGCACTTGA
	Probe	CACTTCCCGGGTGATGCTCAGCA
PDGFβ	Forward primer	CGGACGGTGCGAATCC
	Reverse primer	TGTCATGGGTGTGCTTAAACTTTC
	Probe	CCGGCCCCCCAAAGGGAAG
VEGF	Forward primer	AGCGGAGAAAGCATTTGTTTG
	Reverse primer	AACGCGAGTCTGTGTTTTTGC
	Probe	CCAAGATCCGCAGACGTGTAAATGTTCC
HIF1α	Forward primer	CCCAGCTGTTCACTAAAGTGGAA
	Reverse primer	GCATCGGGCTCTTTCTTAAGC
	Probe	TGAGGACACGAGCTGCCTCTTCGAC
SDF1α	Forward primer	ATCAGTGACGGTAAGCCAGTCA
	Reverse primer	TGGCGACATGGCTCTCAAA
	Probe	CTGAGCTACAGATGCCCCTGCCGA

Abbreviations: BDNF, brain-derived neurotrophic factor; HIF1α, hypoxia-inducible factor-1α; PDGFβ, platelet-derived growth factor; SDF1α, stromal cell-derived factor -1α; VEGF, vascular endothelial growth factor.

### Intrathecal 4-CIN Infusion

To pave the path for future experiments using transgenic and knockout mouse models to gain further insights into the mechanisms of the benefits of KD, we used male C57Bl/6 mice in the following experiment. Animals were randomly assigned into four groups: 1) SD-treated with PBS infusion, 2) KD-treated animals with PBS infusion, 3) SD-treated animals with 4-CIN infusion, and 4) KD-treated animals with 4-CIN infusion. Contusive SCI was performed using the IH-spinal cord impactor (75 kdyne) as described previously at the C4-C5 level as described previously [33]. Immediately after the impact, animals were placed in a stereotaxic frame with its head positioned at ~90° angle to horizontal to expose the cisterna magna. For intrathecal infusion, the polyethylene catheter was stretched to reduce its diameter. After a pre-puncture of the dura mater with a fine needle, the stretched catheter was inserted into the subarachnoid space (catheter tip ending at C2/3 level). The other end of the catheter was connected to an Alzet osmotic mini-pump (flow rate: 0.5 μl/h, model 1007D; Alzet, Palo Alto, CA) filled with either α-cyano-4-hydroxycinnamate (4-CIN; 2 mmol/LC2020, Sigma Aldrich) or 0.9% NaCl-solution. All pumps were primed overnight at 37°C to attain a constant flow rate. The osmotic mini-pumps were implanted into the subcutaneous space at the back of the neck close to the lesion site. The skin incision was closed with sutures. 

Seven days after injury, animals were transcardially perfused with phosphate-buffered saline (PBS) followed by 4% paraformaldehyde (PF). The spinal cord containing the lesion were dissected, post-fixed in PF overnight, cryoprotected in increasing concentrations of sucrose solution (12%, 18%, 24%), and frozen in tissue-Tek. The lesion block was cryostat sectioned in a traverse orientation, i.e. cross sections, at a thickness of 20 μm). 

Lesion areas of longitudinal sections were based on the disruption of the cytoarchitecture using GFAP immunostaining. Total lesion volume (mm^3^) was calculated with the Cavalieri's Estimator of Morphometric Volume on 7x 20 μm thick tissue sections spaced 200 μm apart through the dorso-ventral axis of the spinal cord: [SUM lesion area * distance between sections]-[lesion area epicenter * section thickness] [31]. To approximate the size of the lesion cavity, the edge of the cavity was manually traced and the area enclosed by the trace was quantified in mm^3^. 

### Statistics

All data are presented as the mean ± the standard error of the mean (SEM). The data was analyzed using an ANOVA repeated measures, Student’s t-test or Chi-square test. Statistical significance was set at p≤0.05. 

## Results

### Food intake & bodyweight following KD feeding are similar to SD rats

**Figure 1:**
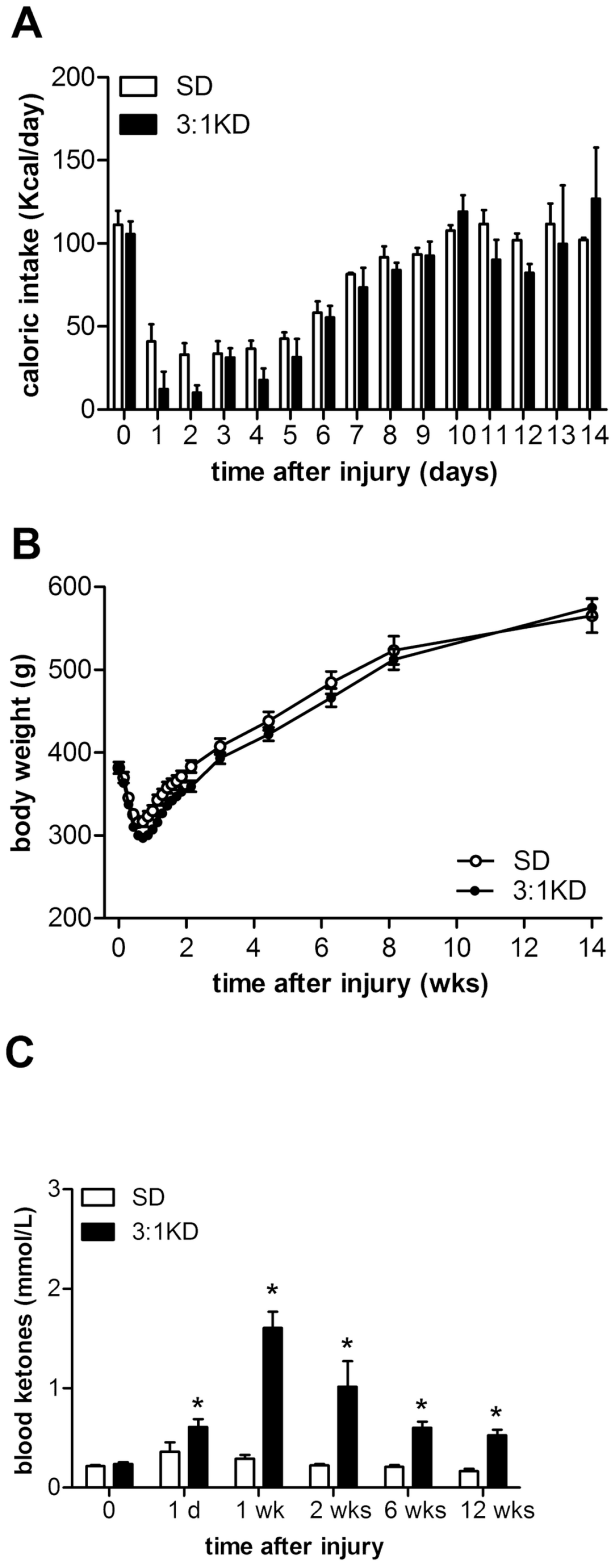
Body weight, caloric intake, and blood ketone levels in rats consuming a 3:1 ketogenic diet (KD) or standard diet (SD) after C5 hemi-contusion injury (experiment 1). (A) During the first week both groups of rats showed reduced food intake but this was more pronounced in the 3:1 KD group on day 1 and 2, which (B) resulted in some additional transient weight loss compared to SD animals. After 2 weeks until the remainder of the experiment, there was no difference in body weight observed between the groups. (C) Blood ketone (ß-hydroxybutyrate) levels increased when consuming 3:1 KD. Error bars indicate SEM. * p ≤ 0.05 (ANOVA repeated measures).

Both KD and SD rats showed less food intake during the first week after injury, except that the KD animals ate even less on day 1 and 2 than the rats on SD, likely because they had an aversion to a novel diet. Within one week following injury, food intake recovered in both SD and KD-fed animals (Figure 1A). By 2 weeks, body weight of both groups had returned to pre-injury levels and continued to increase similarly thereafter. Following 14 weeks of KD-feeding the bodyweight was similar to that observed for SD-fed rats (Figure 1B). 

### KD-induced increase in blood ketone levels peaked at 1 week

Blood ketone levels of the KD group were significantly higher than in SD animals (Figure 1C), reaching maximal levels around 2 mmol/l by 1 week post-injury (*t*(18)=6.489, p<0.001). After week 1, ketone levels slightly decreased in KD treated animals, but were still significantly higher compared to the SD group (*t*(16)=4.902, p<0.001). 

### KD-fed rats showed a larger forepaw range of motion during grooming

**Figure 2:**
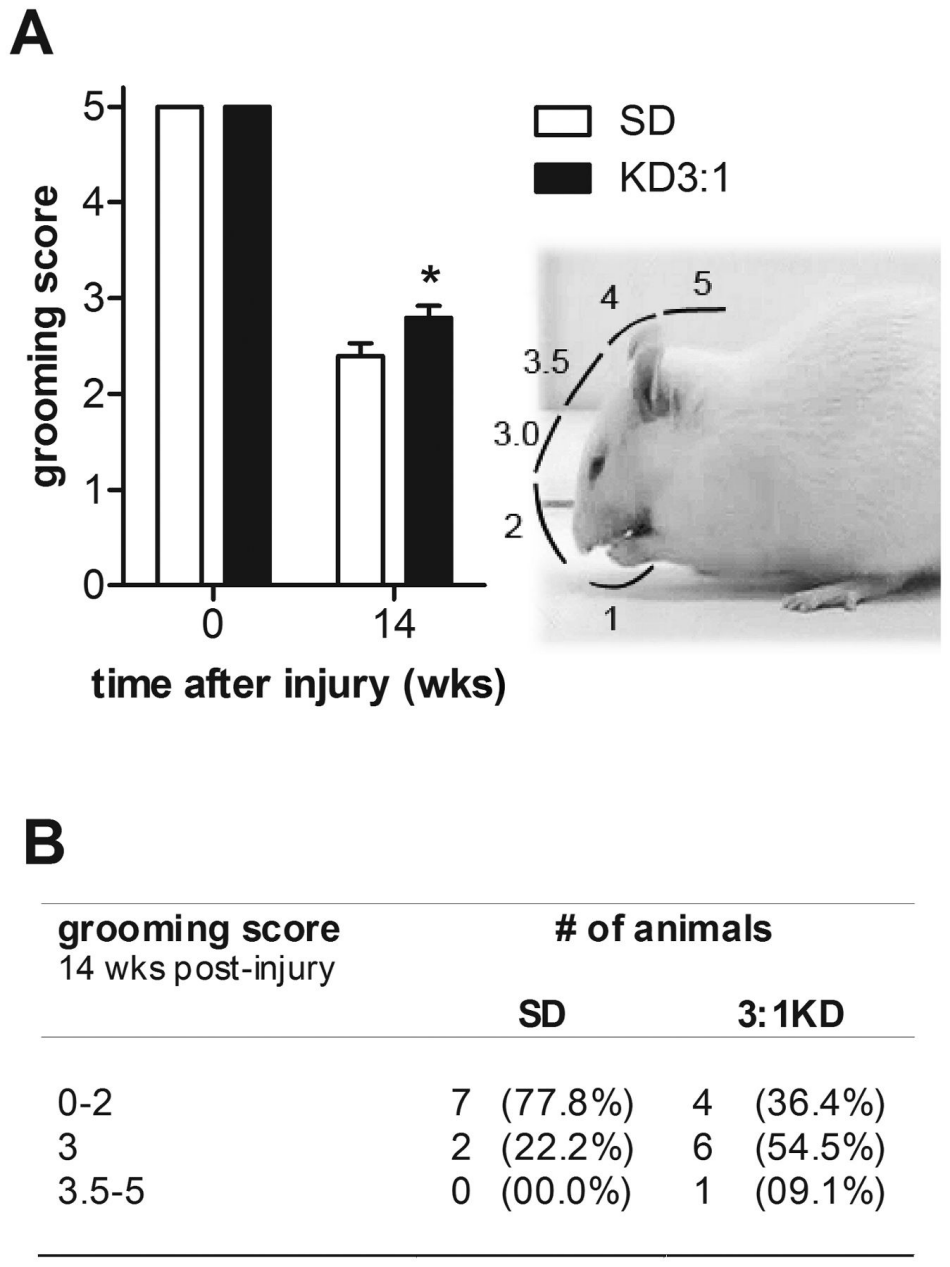
Effect of 3:1 ketogenic diet (KD) on forelimb usage during grooming after C5 hemi-contusion (experiment 1). A. Scoring system used: Animal’s forepaw 0) was unable to contact any part of the face or head; 1) touched underside of chin and/or mouth area; 2) contacted area between nose and eyes, but not the eyes; 3) contacted eyes and area up to, but not including, the front of ears; an additional 0.5 score was given if the paw reached higher than half of the areas total length; 4) contacted front but not back of ears; 5) contacted area behind ears. (B) 3:1 KD treated rats demonstrated an increased grooming score, compared to SD animals. (C) After 14 wks of 3:1 ketogenic diet treatment (KD), we observed an improved forelimb range of motion compared to standard diet fed animals (SD). 66% of the KD animals reached up to the level of the eye (score 3). Most of the SD animals (88%) could only reach as far up as the nose (score 2). Error bars indicate SEM. * p=0.05 (Chi-square test).

Grooming is an innate behaviour that comprises different type of movements such as postural adjustments and forelimb strokes over the entire face, often reaching to behind the ears. Functional use of the forelimbs was evaluated by examining the active range of motion of each forelimb during grooming. KD rats showed significantly higher grooming scores compared to SD animals (p=0.051; Figure 2A-B). The majority of animals in the KD group (7 out of 11; 63.6%) were able to raise their affected paw up to eye level (score 3), whereas most of the SD animals (7 out of 9; 77.8%) could at best reach as far as the nose (score 0-2; Figure 2C; p=0.064, Pearson Chi-square). This finding was robust, as we found in an additional KD-experiment a similar improvement in the range of motion of the ipsilateral forelimb (p=0.029, Pearson Chi-square; Table 5).

**Table 5:** Number of animals for each grooming score of rats fed SD or 3:1 KD after SCI. The numbers between brackets represent the percentage of animals.

**Grooming Score**	**SD**	**3:1KD**
*Time point: 11 wpi*		
0-2	0 (00.0%)	1 (05.9%)
3	12 (66.7%)	4 (23.5%)
3.5-5	6 (33.3%)	12 (70.6%) *
*Time point: 12 wpi*		
0-2	1 (05.6%)	1 (05.9%)
3	12 (66.7%)	6 (35.3%) *
3.5-5	5 (27.8%)	10 (58.5%)

* Significant different from SD (p=0.029); Pearson chi-square

### Skilled forelimb reaching recovered better following KD feeding

Humans with cervical spinal cord injuries, while still able to breath independently, expressed a strong desire to regain hand function, as this would allow allows for a greater degree of independence and quality of life [34]. Reaching for objects in rodents is deemed to model some aspects of hand function [35]. The Montoya staircase test for reaching success represents a convenient tool for the bilateral measurement of an animal's forelimb extension and grasping skills. 

**Figure 3:**
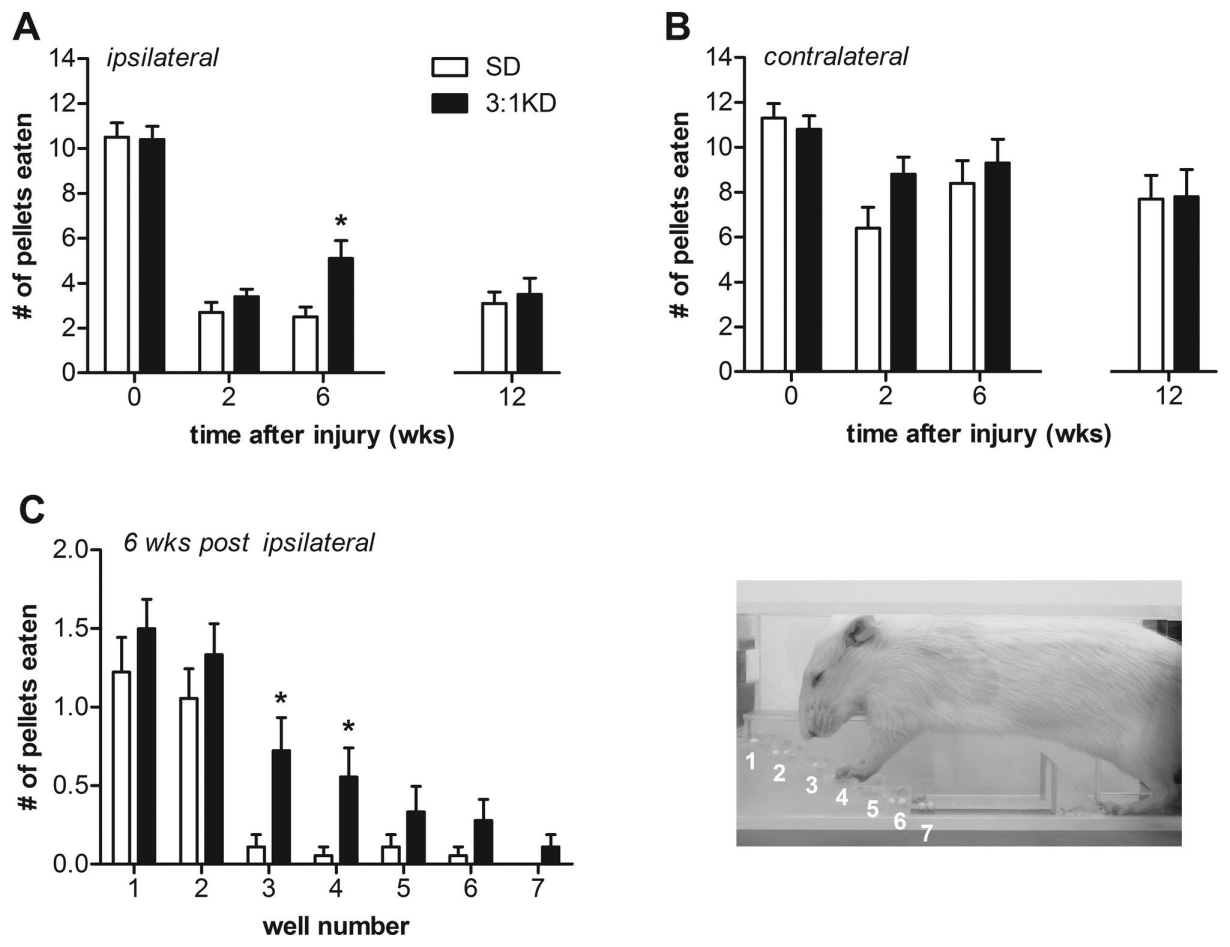
3:1 ketogenic diet (3:1 KD) improved food pellet reaching as assessed with the Montoya staircase test (experiment 2). (A) C5 hemi-contusion injury produced marked impairments in reaching with the ipsilateral forelimb, as illustrated by decreased ability to retrieve pellets in the staircase test. However, at 6 wks post-injury 3:1 KD treated animals retrieved pellets twice as successful with the ipsilateral paw compared to animals fed SD. (B) Animals also displayed a decreased skilled reaching success with the paw contralateral to the lesion. No differences were observed between groups. (C) KD animals were more successful than SD animals in retrieving pellets from the lower wells. Error bars indicate SEM. * p ≤ 0.05 (t-test).

**Figure 4:**
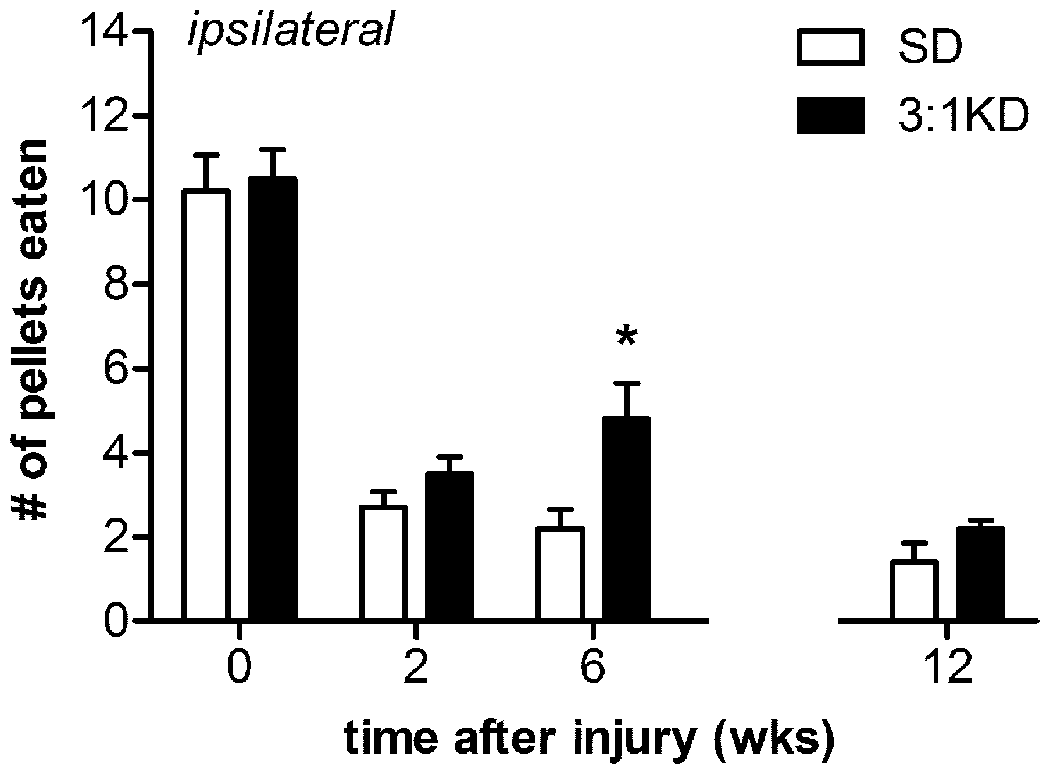
3:1 ketogenic diet (3:1 KD) improved reaching as assessed with the Montoya staircase test in an independent set of rats: experiment 3. At 6 weeks post-injury, 3:1 KD fed animals showed an increased success to retrieve pellets in the staircase test compared to standard diet (SD) rats. This beneficial effect however was lost at 12 weeks post-injury. Error bars indicate SEM. * p ≤ 0.05 (t-test).

In the Montoya staircase test animals reach from a central platform with their forelimbs to retrieve food pellets from eight descending steps with shallow wells [26,27]. The pellets in lower wells are more difficult to grasp than those in wells higher on the staircase. KD-fed rats exhibited greater reaching success with the ipsilateral paw compared to SD animals at 6 weeks post-injury (*t*(1,34)=2.640, p=0.012; Figure 3A), with no differences were observed for pellet retrieval with the contralateral paw (Figure 3B). When using the ipsilateral paw, KD-fed animals were able to retrieve significantly more number of pellets from well number 3 and 4 compared to SD animals (ANOVA repeated measures, *F*(1,34)=6.971, p=0.012; well#3, *t*(34)=2.725, p=0.010; well#4, *t*(34)=2.592, p=0.014, respectively; Figure 3C). It is noteworthy here that analysis of the video recording revealed that rats were able to use their tongue to retrieve pellets from the top two steps. Reaching is therefore only considered successful if the pellets were eaten from the bottom five steps (well 3-8). Somewhat surprisingly, the improved retrieval success with the ipsilateral paw was lost when tested at 12 weeks post injury (Figure 3A). Again this finding was robust, as in an additional KD-experiment we found a similar improvement in staircase performance at 6 weeks post-injury (*t*(25)=3.727, p=0.001; Figure 4) which again was lost when followed up at a later time point (Figure 4). There is a possibility that animals could potentially adopt a variety of whole body or postural compensatory strategies in order to obtain food pellets. These compensatory strategies, might have partially masked the differences between both groups at the later time points. An alternative paw-reaching paradigm such as the Whishaw single pellet reaching test, in which the exact nature of the reaching movement is broken down into several defined components, may prove advantageous in detecting reaching deficits.

**Figure 5:**
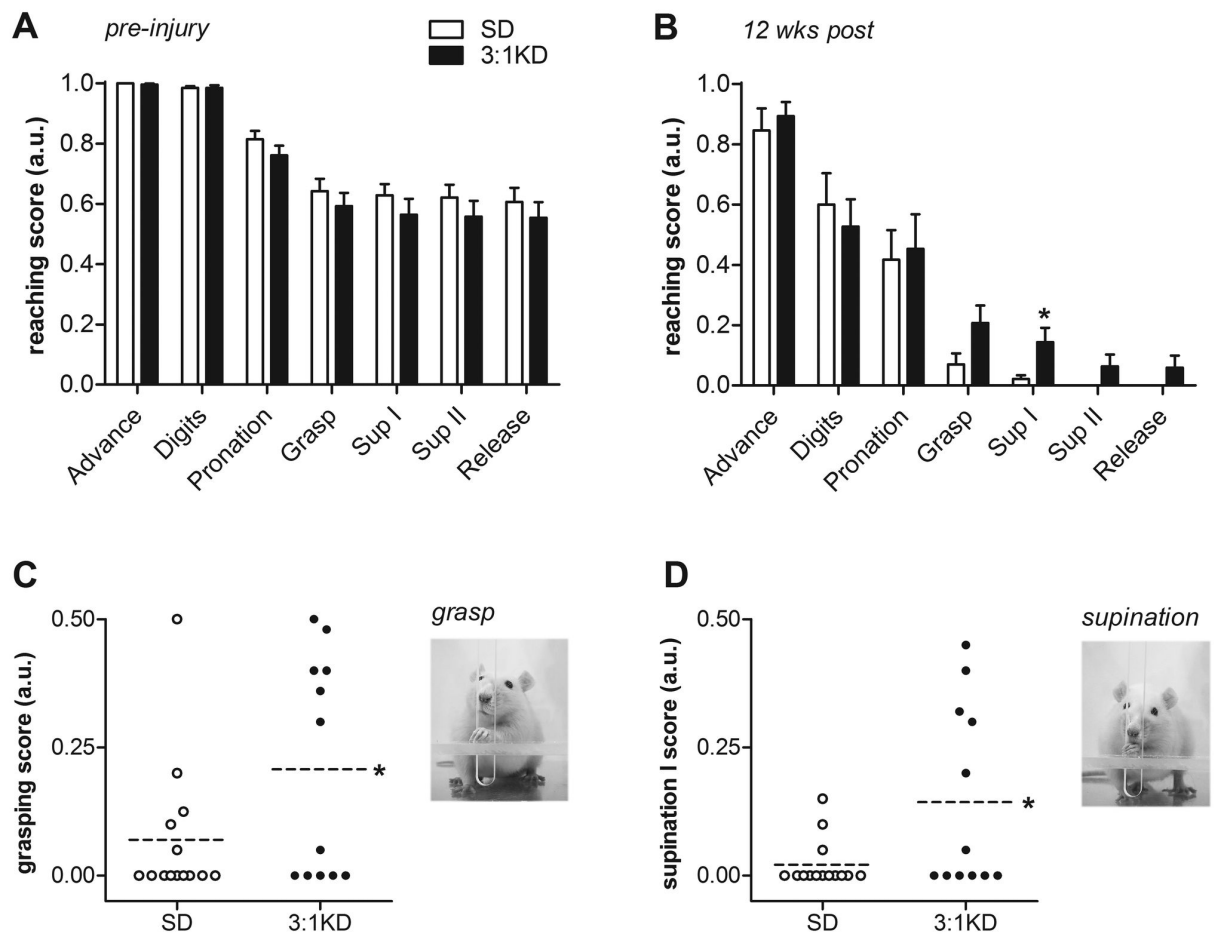
Ketogenic diet (3:1 KD) improved the grasping and supination component of the pellet reaching movement (experiment 2). (A-B) The following components were scored: I) Advance; II) Digit extension; III) Pronation; IV) Grasp; V) Supination I; VI) Supination II; VII) Release. On a 3-point scale from 0-1 a score of 1 indicated the presence of the “correct” or “complete” movement, 0.5 the presence of an irregular or incomplete movement and 0 the absence of the movement. (A) Compared to pre-operative scores, (B) C5 hemi-contusion injury dramatically affected reaching components in both SD and KD group. (CD) Grasping and (EF) supination I results of individual animals are blotted. Post-injury KD treatment for 12 weeks resulted in higher average (C) grasping and (E) supination scores based on 10 reach attempts. Dotted line indicates the average score per group. Besides the average score, we also analyzed the highest score achieved (i.e max score) of each animal to assess the animal’s best movement capability during grasping and supination I. (D) None of the SD animals displayed normal grasp movements, however, 50% (6 out of 12) 3:1 KD animals showed a max score of 1 at least once during 10 reach attempts. (F) Despite the severe deficit in supination, almost 3-times as many KD animals (5 out of 12) were capable of normal supination movements compared to SD animals (2 out of 14). Error bars indicate SEM. * p=0.021, ** p=0.003, # p=0.06 (t-test).

 It has recently been shown that rodent digit use during grasping actions are very similar to that described for primates [35]. Reaching chambers like the ones used in the Whishaw test allow for a detailed analysis of the components of the reaching movements [29,30] and have been useful for the investigation of skilled forepaw use and motor functioning deficits after unilateral spinal cord damage in rodents [30]. The injury affected the animals’ ability to grasp food pellets and to pronate the paw, while supination II and release of the food pellet were basically abolished (Figure 5A-B). KD treatment, however, resulted in higher average scores for the components grasping for food (*t*(24)=1.974, p=0.060; Figure 5C) and supination I (*t*(24)=2.464, p=0.021; Figure 5D). To assess their best movement capability of the different reach components, we additionally analyzed the highest score achieved during 10 separate reaches. Significantly more KD animals (50%, 6 out of 12) achieved maximal grasping scores of 1, reflecting their enhanced ability to perform normal grasping movements. In contrast, none of the animals in the SD group reached a score of 1 (0%, 0 out of 14; p=0.003, Pearson Chi-square). Interestingly, while the success on the Montoya staircase was similar at 12 weeks post injury, the quality of the reaching movement was still significantly better at 12 weeks in the animals on KD as revealed by the Whishaw test. 

### KD-fed rats showed persistently greater forelimb usage during cylinder rearing even when switched back to SD

**Figure 6:**
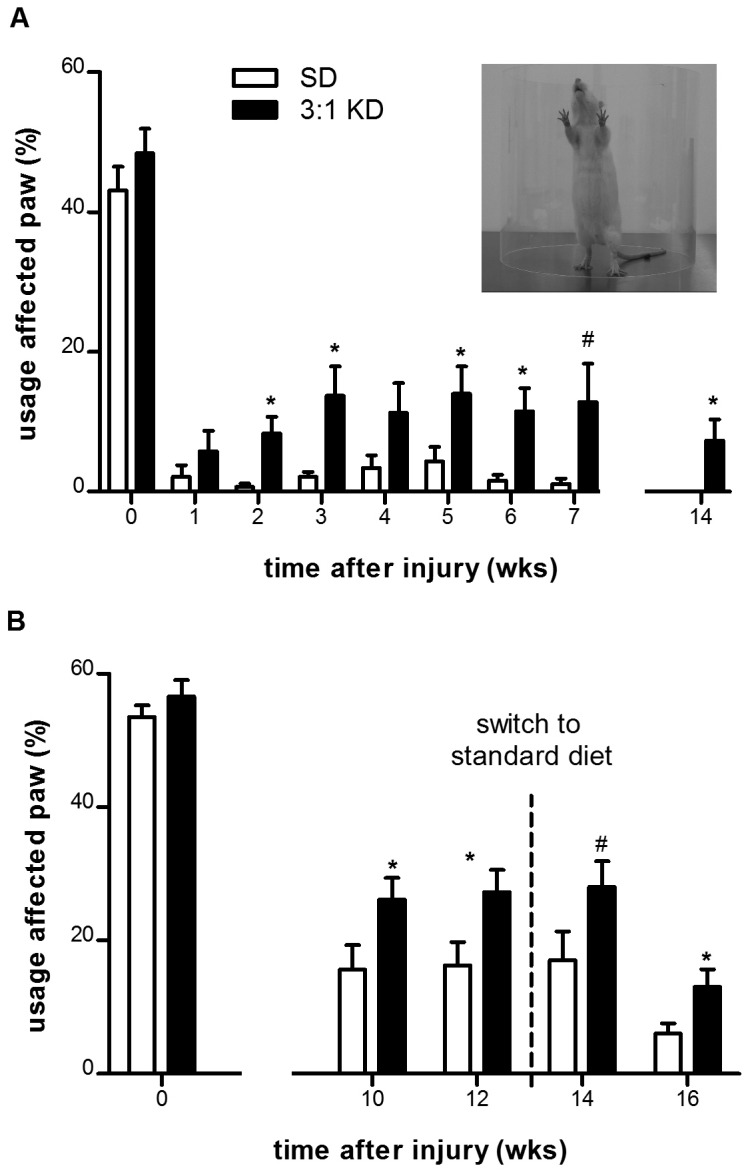
Forelimb usage improved under ketogenic diet (3:1 KD) treatment in C5 hemi-contused rats (experiment 1). (A) The contusion injury produced marked deficits in rearing performance in both groups. Rats fed a 3:1 KD showed increased paw placements of their ipsilateral paw when placed simultaneously with the contralateral paw (both). (B) In a separate cohort of rats (experiment 2) we demonstrated that after switching from to a standard carbohydrate-based diet after 12-weeks of 3:1 KD treatment the forelimb usage remained elevated for 4 weeks compared to SD animals. Error bars indicate SEM. * p ≤ 0.05, ^#^ p=0.06 (t-test).

The cylinder test provides a way to evaluate a rodent's spontaneous forelimb use. Ipsilateral forelimb usage during vertical exploration in uninjured healthy rats is usually around 45% of total wall placements. After hemi-contusion injury, SD rats used their injured forelimb less than 3% of the time while rearing (Figure 6A). KD animals however, exhibited a four-fold increase in use of the impaired forelimb to around 13% (ANOVA repeated measures, *F*(1,18)=7.907, p=0.012). This improvement in paw usage was observed 2 weeks after starting the treatment (*t*(18)=2.884, p=0.010) and was sustained throughout the study (14 weeks post-injury; *t*(18)=2.213, p=0.040; Figure 6A).

In an additional, independent group the beneficial effect of KD on forelimb usage was reproduced (ANOVA repeated measures, *F*(1,27)=6.020, p=0.021, Figure 6B), demonstrating improved usage of the affected paw after 10 and 12 weeks of KD treatment (wk10: *t*(31)=2.114, p=0.043; wk12: *t*(30)=2.300, p=0.029). Importantly, after switching back to SD for an additional 4 weeks, the improvement was sustained for the remainder of the study (wk14: *t*(32)=1.888, p=0.068; wk16: *t*(31)=2.590, p= 0.014; Figure 6B). 

### KD promotes gray matter sparing

**Figure 7:**
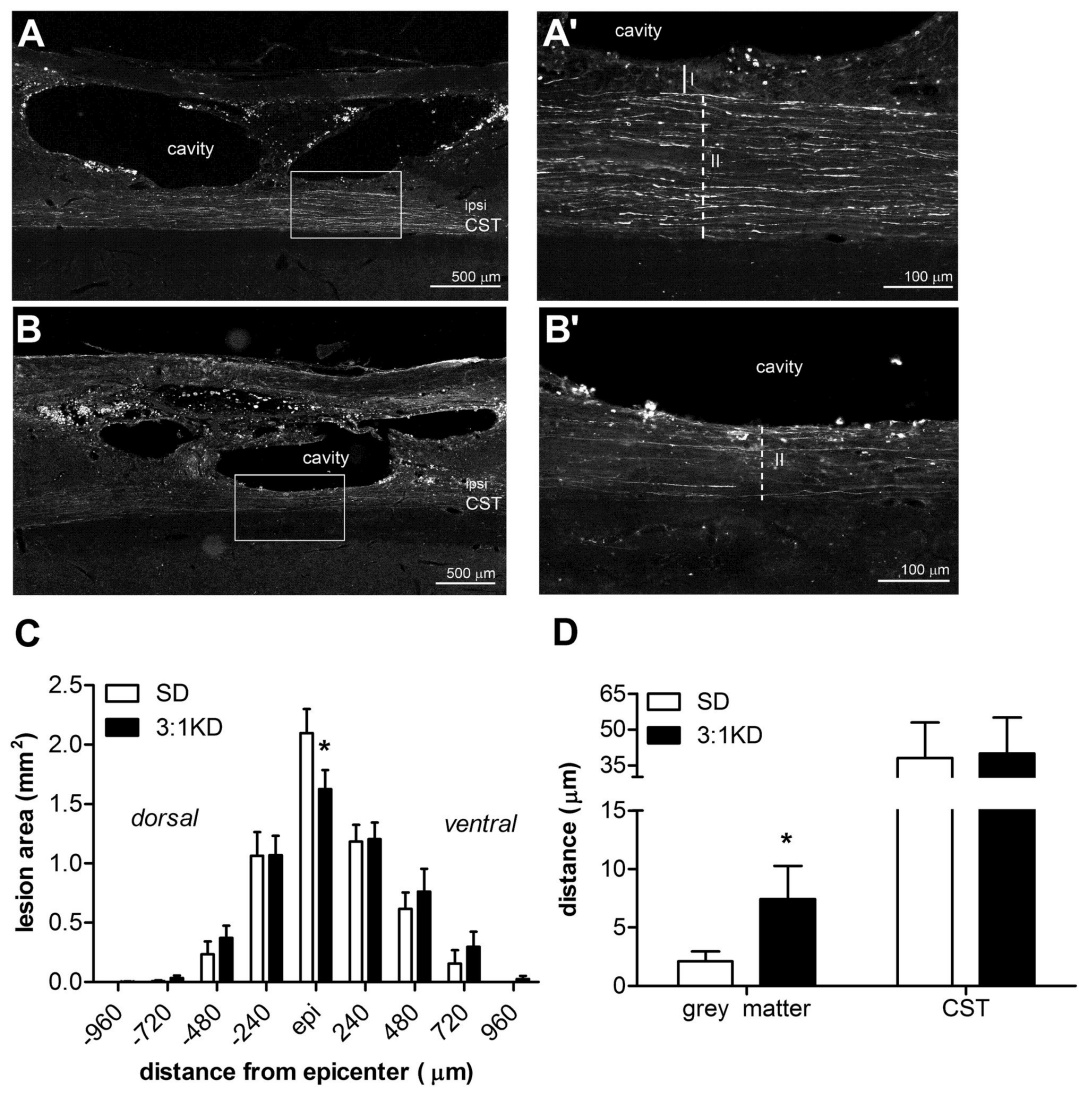
Ketogenic diet treatment (3:1 KD) is neuroprotective after C5 hemi-contusion (experiment 1). (A-B) Representative pictures of emerald traced corticospinal tract (CST) axons 14 wks post-injury (Zeiss Axioplan fluorescent microscope). A higher magnification of the box in figure A and B is shown in respectively A’ and B’, scale bar: 500 μm. (A’) Grey matter and (B’) CST sparing measurements, scale bar: 500 μm. Solid line I: length grey matter, sparing dashed line II: length ipsilateral CST sparing. (C) The lesion area at the epicenter and (D) spared grey tissue was significantly increased in spinal cords of KD fed animals compared to the standard diet group (SD). Error bars indicate SEM. * p=0.026, # p=0.030 (t-test, one tailed).

The rostro-caudal extent of the lesion was similar between both groups (SD: 3.5 ± 0.12 mm; KD: 3.63 ± 0.27 mm). Notably, KD treatment reduced lesion area at the epicenter by ~20% (*t*(14)=2.051, p=0.030, one tailed; Figure 7). Total lesion volume, as calculated using the Cavalieri’s Estimator of Morphometric Volume, was not significantly different between the 3:1 KD group (1.40 ± 0.29 mm^3^) and SD group (1.54 ± 0.26 mm^3^).

 Whereas half of the animals showed CST damage throughout the entire dorsal-ventral depth of the CST, the remainder of animals showed some sparing of the CST with some preservation of gray matter (typically ventrolaterally to the dorsal CST). Interestingly in these animals (n=4 out of 8 for both groups), KD treated rats showed ~2.7 fold increase in grey matter sparing compared to SD treatment (*t*(6)=-2.937, p=0.026; Figure 7D). No significant difference in white matter sparing was observed between groups. 

### KD does not enhance corticospinal tract plasticity

While there has been far less work on the effect of KD on neuroplasticity as compared to neuroprotection there are some molecular changes that indicate a potential of increased plasticity with KD. For example, KD increases serum leptin levels by as early as 5 days after starting the diet [36]. Leptin is known to be not only neuroprotective, but it also enhances neuronal plasticity in vitro and in vivo [37-42]. Another known molecular mechanism for synaptic plasticity is the AMP-activated protein kinase (AMPK), which is activated with various stresses including glucose deprivation as would occur on a KD [43,44]. Bcl-2, a protein that controls the mitochondrial membrane transition pore, not only prevents apoptosis but may also promote in vivo regeneration in the CNS [45-47]. A 3 week pretreatment with KD or a 4 day infusion of beta-hydroxybutyrate (β-HB) resulted in an increase in bcl-2 levels in the brain [18], and thus could potentially be another mechanism to improve axonal plasticity after SCI. Finally, the stimulating effect of KD on mitochondrial density [48]could induce neuronal plasticity. Dendritic mitochondria have been implicated in synapse formation following hippocampal stimulation and the number of mitochondria is correlated with the number of newly formed synapses [49-51].

These four potential pathways mentioned above provide a rationale to also assess whether KD increases plasticity/regeneration after SCI. We anterogradely traced the left corticospinal tract projecting to the ipsilateral gray matter of the spinal cord. When comparing the C5/C1 ratio within 1 mm rostral (SD: 106.2 ± 20.8; KD: 83.7 ± 16.3, p=0.42) and 2mm caudal (SD: 113.8 ± 47.7; KD: 98.2 ± 31.1, p=0.79) from the lesion boundary, we found no significant differences between groups. 

### There was no detectable increase in BDNF, VEGF, PDGFβ or SDF1α

A previous report by Puchowicz et al [18] showed increased expression/stabilization of Hypoxia Inducible Factor -1α(HIF1α) in rats subjected to focal ischemia after consuming a KD for 3 weeks. We also observed HIF1α immunoreactivity in the lesion penumbra most prominently – but not exclusively - associated with astrocytes (not shown). HIF1α can activate numerous downstream effectors such as brain-derived neurotrophic factor (BDNF), vascular endothelial growth factor (VEGF), and stromal cell-derived factor-1α (SDF1α) [52]. In the context of spinal cord injury, these molecules act not only on creation of new vasculature but also are involved in neurogenesis [52-55]and axonal plasticity in the injured spinal cord [56]. 

To evaluate if the behavioral and neuroprotective benefits of KD were associated with increased growth factors and neurotrophins in the injured spinal cord, we measured the level of mRNA between BDNF, VEGF, platelet-derived growth factor-β (PDGFβ), HIF1α and SDF1α. Spinal cord samples (n=4 per group) taken at day 14 after SCI showed a similar level of mRNA expression for all genes examined (Table 6), and no significant differences between 3:1 KD and SD were observed. 

**Table 6:** Relative gene expression of BDNF, PDGFβ, VEGF, HIF1α, SDF1α in spinal cord of rats fed 3:1KD.

	**BDNF**	**PDGFβ**	**VEGF**	**HIF1α**	**SDF1α**
**KD/SD**	0.65 ± 0.29	1.54 ± 0.78	1.03 ± 0.37	1.29 ± 0.37	3.15 ± 1.46

The expression levels of mRNAs were measured by real-time PCR. Values represent relative expression levels of genes in spinal cord of KD rats (n=4) compared with control SD rats (n=4). Abbreviations: BDNF, brain-derived neurotrophic factor; HIF1α, hypoxia-inducible factor-1α; KD: ketogenic diet; PDGFβ, platelet-derived growth factor-β; SD: standard diet; SDF1α, stromal cell-derived factor - 1α; VEGF, vascular endothelial growth factor. Data are mean ± SEM.

### KD increased the expression of glucose and monocarboxylate transporters

**Figure 8:**
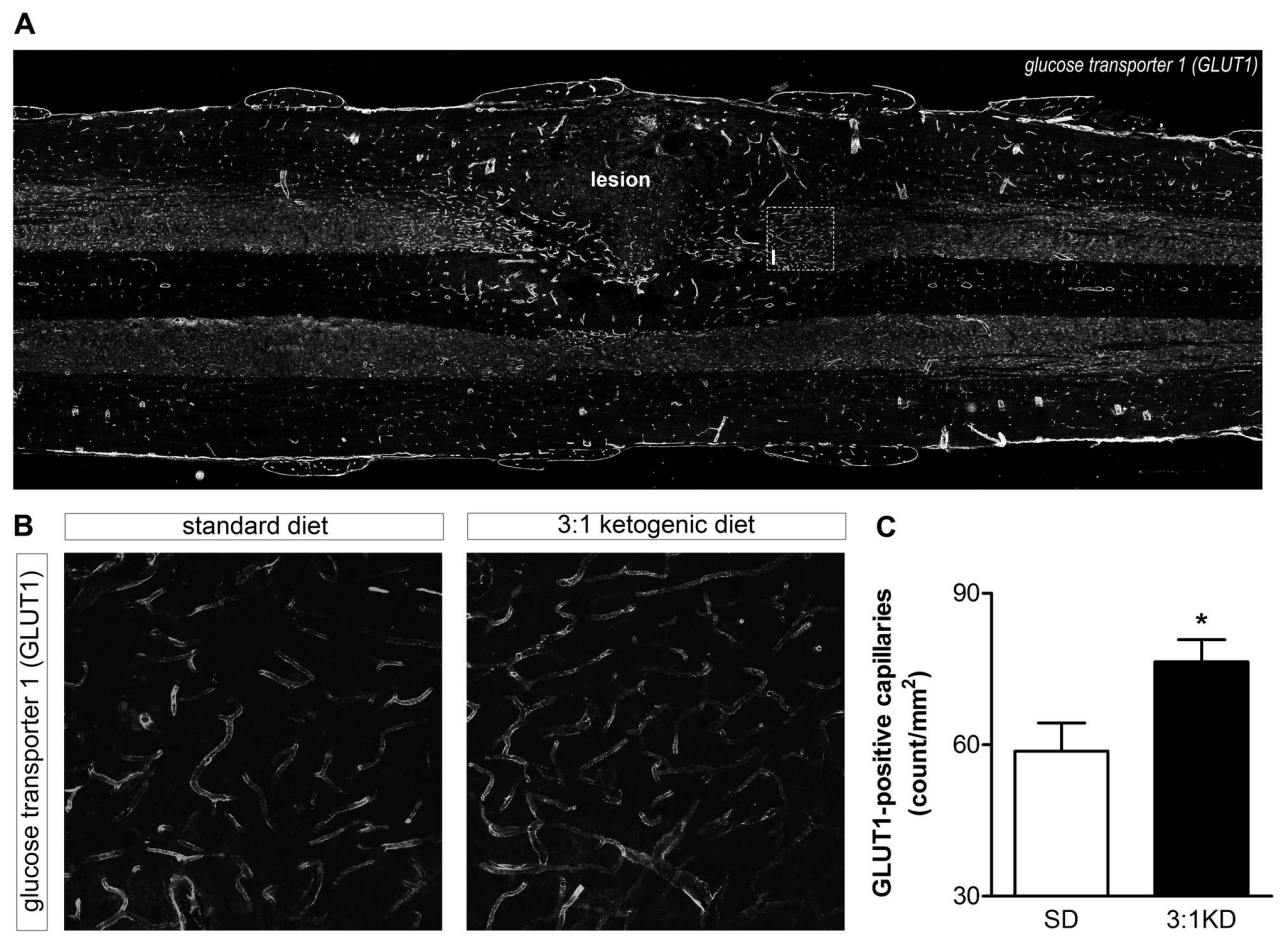
Glucose transporter-1 (GLUT1) immunoreactivity increased in the capillaries of the lesion penumbra in rats on ketogenic diet. (A) Representative pictures of spinal cord section showing GLUT1 staining characteristic of blood vessels at 7 days following injury (5x magnification; Leica DM5000B microscope, Leica Microsystems). Note the enhanced GLUT1 staining at the penumbra of the lesion cavity. To count number of Glut1 positive capillaries a 750×750 μm window was placed over the gray matter, with bottom of the window always touching boundary between gray and white matter (represented by box I). Scale bar: 500 μm (B) Higher magnification of representative field for GLUT1 quantification analysis of the SD and KD group, scale bar: 100 μm. (C) Blood vessel density, as measured by GLUT1 staining, increased by ~30% in the KD group compared to the SD group. Error bars indicate SEM. * p ≤ 0.05 (t-test).

The glucose transporter-1 (GLUT1) is a major glucose transporter restricted to the vascular endothelial cells. Expression of GLUT1 immunoreactivity was increased in the penumbra surrounding the lesion area, whereas away from the lesion low expression of GLUT1 was detected by blood vessels (Figure 8A). KD treatment leads to a significant increase in number of GLUT1-positive blood vessels by 30% in the grey matter adjacent to the lesion border (*t*(7)=2.831, p=0.049; Figure 8B-C). The number of RECA-positive blood vessels did not differ between groups (SD: 118.7 ± 3.6; KD: 112 ± 2.9; *t*(7)=0.79, p=0.19). 

**Figure 9:**
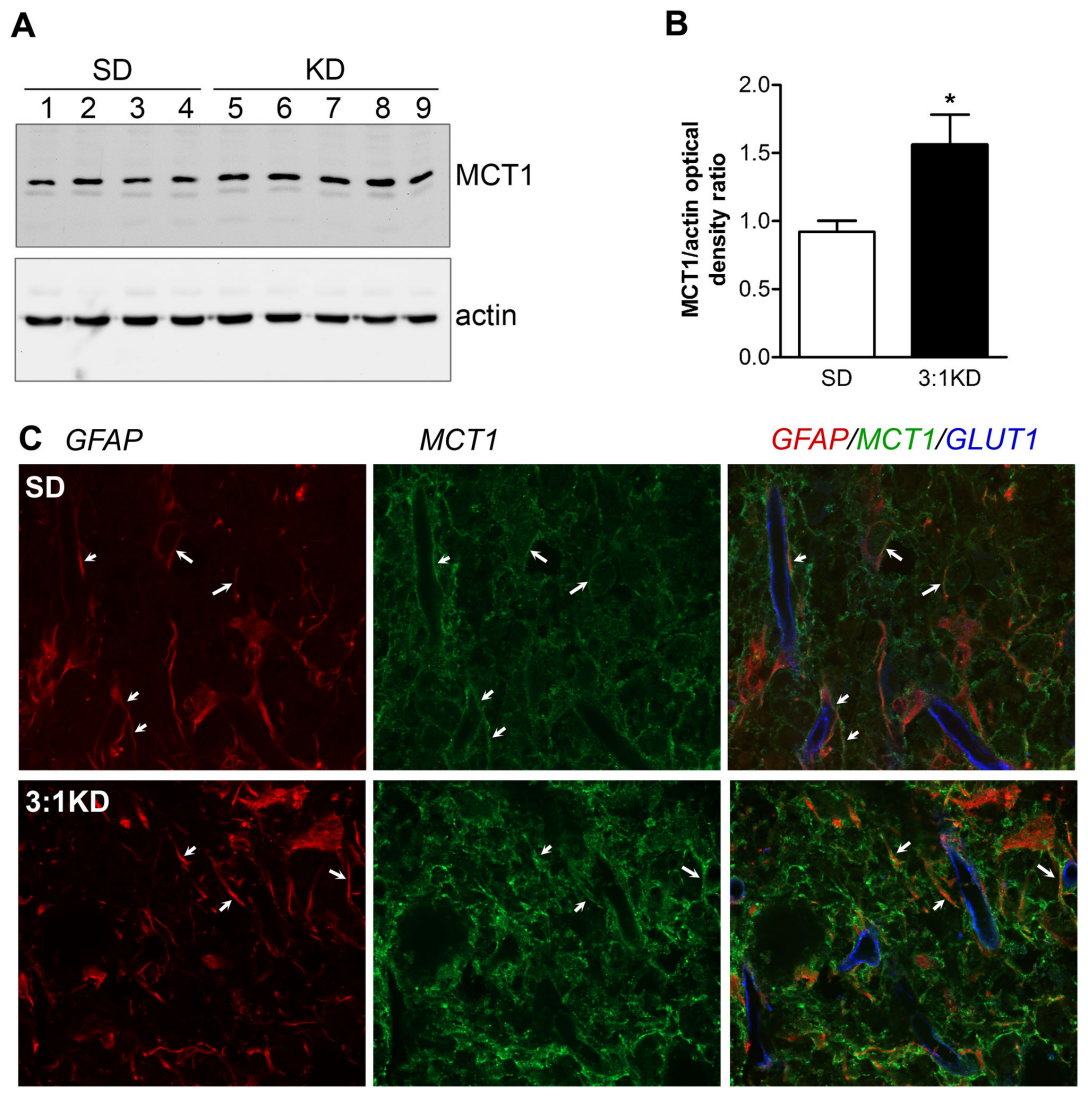
Monocarboxylate transporter-1 (MCT1) expression increased in spinal of KD fed rats at 14 days after SCI. (A) Immunoblot of spinal cord samples probed with antibody to MCT1 showing the expected 42 kDa MCT1 band. As a control for equal protein loading, the corresponding actin signal is shown. (B) The relative MCT1 expression level was significantly higher (1.7-fold) in the KD group compared to the SD group (average of 4 rats). Values are normalized to actin values. Error bars indicate SEM. * p=0.021 (t-test). (C) Triple immunofluorescence confocal image to characterize the localization of MCT1 (green) on glial fibrillary acidic protein (GFAP, red) and glucose transporter-1 (GLUT1; blue) in the spinal cord of a KD fed rat (Spinning disc confocal microscope, 63x, 1.4 NA).

Availability of ketone bodies for neuronal uptake is highly dependent on transport of these substrates through Monocarboxylate transporter-1 (MCT1) [57]. Compared to spinal cord samples of SD rats, MCT1 protein expression was significantly increased by ~2-fold in the KD group (*t*(7)2.473, p=0.021; Figure 9A-B). This KD-induced upregulation of MCT1 expression was primarily observed in the gray matter near the lesion penumbra (Figure 9C). Confocal imaging revealed punctate immunoreactivity throughout the neuropil of the spinal cord, including a pronounced co-localisation with GFAP-positive astrocytes (Figure 9C).

### Inhibition of MCT1 abolishes KD-induced tissue protection

**Figure 10:**
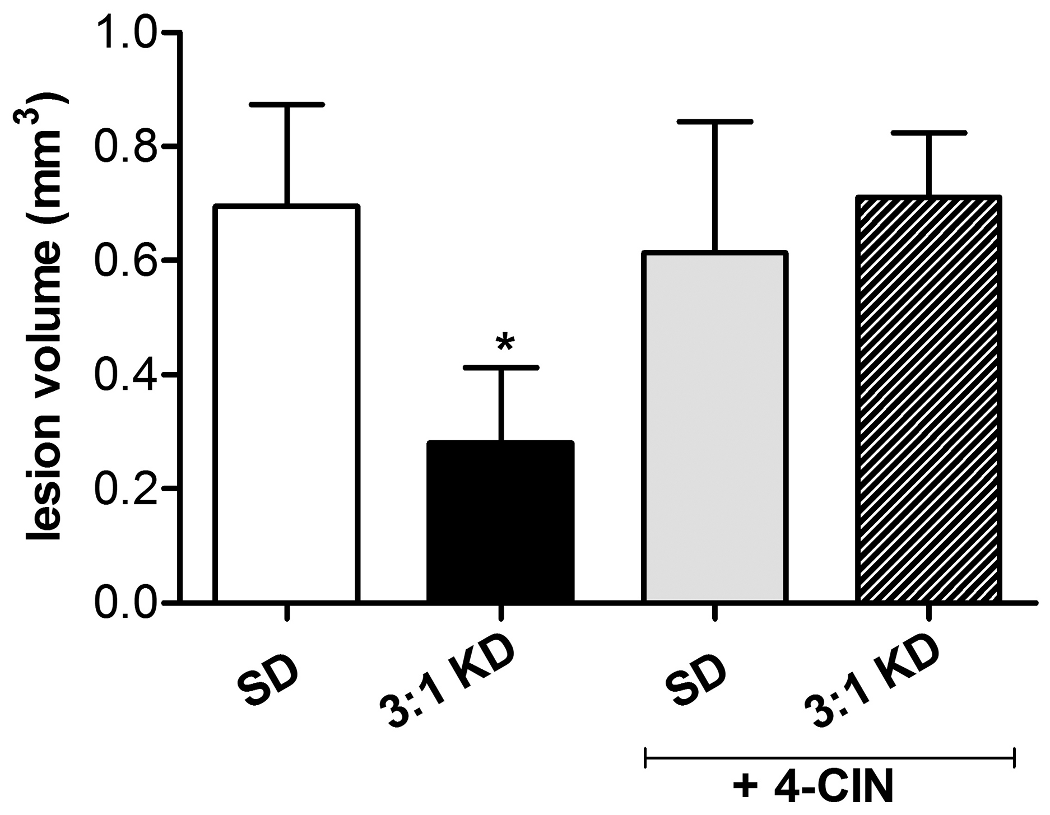
KD-induced neuroprotective effect is lost in when MCT transport is inhibited using α-cyano-4-hydroxy-cinnamate (4-CIN). At day 7-post injury, the total lesion volume (Cavalieri’s Estimator of Morphometric Volume) was decreased in spinal cords of KD fed animals compared to the standard diet group (SD). However, no difference in lesion volume between both groups was observed when animals were treated for 7 days with the MCT inhibitor 4-CIN. Error bars indicate SEM. # p=0.057 (t-test, one tailed).

To examine the impact of blocking MCT activity on KD-induced neuroprotection, spinal cord injured mice were intrathecally infused with α-cyano-4-hydroxycinnamate (4-CIN) (Figure 10). In agreement with our rat data, vehicle-infused KD mice demonstrated a decrease in lesion area relative to vehicle-infused SD controls (*t*(7)=1.778, p=0.057, one tailed). This neuroprotective effect; however, was lost when KD-treated animals were intrathecally infused with 4-CIN and resulted in no significant differences when compared to the 4-CIN-infused SD group (*t*(10)=0.494, p=0.316). 

## Discussion

We demonstrated for the first time that post-injury treatment of KD with a 3:1 ratio of fat to carbohydrates and proteins promoted neuroprotection and improved neurologic outcomes following spinal cord injury (SCI); here a model of cervical hemicontusion. The robustness of this therapeutic effect was emphasized by the observation of these improvements in multiple replications. Importantly, we demonstrated that after returning to a standard diet after 12 weeks of consuming KD, the functional benefits were maintained. Besides improved behavioral recovery, KD treatment reduced lesion size and increased grey matter sparing. While it is likely that KD modulates a broad spectrum of targets, in this study we revealed that KD increased GLUT-1 positive blood vessel density and expression of the MCT1. Furthermore, we demonstrated that blocking MCT transport by 4-CIN abolished the neuroprotection by KD. 

 We initiated KD not until 4 hours after injury and food intake was low for 2 days. This time window of intervention differs from the majority of drug treatments for SCI, which have been studied with only little to no delay [58,59]. For most treatments only a 30-min time window of intervention has been established thus far, which unfortunately can only be achieved in a small subpopulation of SCI patients. By this time window criterion KD is one of most effective treatments and hence the translation of KD to humans seems feasible. 

 In contrast to a high fat diet used in obesity research, the 3:1 KD used in our study is a very low carbohydrate, high fat diet; while many high-fat diets used in obesity laboratory animal research contain a decreased fat to carbohydrate ratio (i.e. increased carbohydrate content). In our study both groups had *ad libitum* excess to either 3:1KD or SD. Spinal cord injured rats fed a 3:1KD diet containing 6.76 kcal/g reduced the amount of food they ate to equal the caloric intake of rats fed SD containing 3.79 kcal/g. Body weight was therefore not affected by the diet intervention. Interestingly, when we treated animals with a 7:1KD after a mild dorsolateral crush injury, improved paw usage during spontaneous vertical exploration was evident (data not shown); however, this improvement was accompanied by a reduced body weight. We hypothesized that the low protein content in 7:1 KD was inadequate for general growth, given that raising the protein content in the 3:1 KD prevented weight loss. The absence of significant weight loss or weight gain with the 3:1 KD makes this formulation much more clinically applicable.

The unilateral contusion model used in our study produced marked impairments in skilled reaching with the ipsilateral forelimb, as illustrated by the decreased ability to retrieve pellets in the staircase test. The improvement seen in pellet reaching at 6 weeks post-injury by KD was robust and reproducible in our hands. We surmise that the decline during follow-up assessment could be due to the fact that animals experience limited success while attempting to reach the pellets with the ipsilateral paw, so they choose not to make further attempts. Such “learned non-use” of the ipsilateral forelimb has been reported following stroke in monkeys, rats and humans [29,60,61]. In our study, a similar phenomenon can be observed for both SD and KD fed animals in the cylinder rearing test. Over the time course of the experiment both groups exhibited a decline in general paw usage.

 KD treatment significantly improved reaching components that involve fine manipulation of the distal muscles of the digits and supination. These involve the integration of several motions including paw rotation, continued pellet grasp and coordinated arm withdrawal. This is clinically relevant, since a recent study showed that humans and rats display a similar sequence of digit flexion/extension and supination/pronation movements during reaching for food [35]. Since similarities in fine hand movements exist between rodents and humans, our results would imply that KD may impart improvements in one of the most coveted functions for quadriplegic individuals [34]. 

Others have suggested that some of the benefits of KD are mediated by increased endogenous ketone levels, since the exogenous administration of specific ketones has been proven to be protective in experimental models of Parkinson’s disease, Alzheimer’s disease and glutamate toxicity [62-69]. Interestingly, intravenous infusions of β-hydroxybutyrate (β-HB) administered 1 hour after transient occlusion of the middle cerebral artery ischemia significantly decreases infarct size [70]and similarly exogenous β-HB administration is neuroprotective when administered after a cortical concussion insult in adult rats [71]. 

The diet used in our study is mostly comprised of fats, constituting (65% of the total calories by weight). Fatty acids are known to induce the activity of mitochondrial uncoupling proteins (UCPs), which results in a reduction of the proton gradient across the inner mitochondrial membrane, reducing ATP synthesis, mitochondrial calcium influx, and reactive oxygen species (ROS) production [66,72,73]. Sullivan et al. [73]illustrated the relevance of these observations to the action of KD, demonstrating that mitochondrial UCP activity was increased while ROS formation was decreased in mouse hippocampus after KD treatment. Reduction of calcium influx, and ROS production are major mechanisms of secondary injury following SCI and various agents/health supplements that reduce free radical production have been reported to increase tissue sparing and to improve functional recovery after SCI [74-78]. 

Although their was no obvious difference in blood vessel density as stained by RECA-1 [79], KD treatment did result in a ~30% increase in the expression of glucose transporter-1 (GLUT1) in the vascular system, which has been reported previously in uninjured rats [32]. It is notable, that whereas most Glut-1 vessels were RECA-1-positive, not all RECA-1-labeled vessels co-expressed readily apparent levels of Glut-1. This suggests that KD may affect the regulation of the function of the vasculature, i.e. alter the efficacy of glucose transport across the BBB near the lesion site, however not the extent of spinal cord (re)vascularization after injury. Since the most overt increase of GLUT1 occurred in the gray matter at close proximity to the lesion site of the cord (i.e. penumbra), it is likely that the increased vascular GLUT1 density may reflect local changing metabolic requirements of neuronal cells after injury [80]. Local tissue densities of GLUT1 were found to be closely correlated with local cerebral glucose utilization and transport rate [81,82]. The protective property of KD following SCI may thus be partially associated with restoring the delivery of nutrients to tissue and providing a favorable environment for cell survival and growth. Although we were unable to confirm the reduction in GLUT1 by western blot analysis (data not shown), we suspect that analyzing a large volume of tissue (5-mm spinal cord segment) by during western blotting may have diluted the effect seen in some peri-lesion blood vessels.

 Endothelial cells, astrocytes, neurons and oligodendrocytes are capable of utilizing alternative energy substrates such as lactate, β-HB and acetoacetate. In agreement with others [9,83] we show that KD treatment significantly increased MCT1 expression after 2 weeks, which coincided with changes in blood β-HB levels suggesting increased cerebral uptake of ketones. Our study and others have demonstrated that MCTs are a crucial component of the local energy supply, and the disruption of this transport can exacerbate neuronal damage [84]. MCTs facilitate the transport of monocarboxylic acids such as lactate, pyruvate and ketone bodies across biological membranes. So far, 14 members of MCTs have been identified but only MCT1-4 have been found to be expressed in an active form in the brain and characterized as proton-linked MCTs [85-88]. MCTs have an important role in various tissues due to their function in metabolic homeostasis and their requirement for the transfer of ketone bodies into and out of cells. MCT1 is predominantly expressed by vascular endothelial cells, astrocytes and oligodendrocytes [57,89,90]. Recent evidence supports the notion that, not only the liver, but also astrocytes can produce ketone bodies from fatty acids [91]. Moreover, similar to lactate, ketone bodies might directly protect neurons from death [68] and thus may also play a role in neuroprotection leading to improved recovery of neuronal function after SCI. 

Multiple mechanisms may account for the neuroprotective effects of ketones, which may be in part due to the reduction of neuronal excitation due to several mechanisms including the inhibition of vesicular glutamate transporter by acetoacetate, and increased adenosine levels (for review see 92). In addition, ketone bodies are directly used in the mitochondria for ATP production while glycolysis is bypassed lowering cytoplasmic ATP production, which in turn may increase activity of the ATP-sensitive K^+^ channels and dampen excitation (for review see 92). Since excitotoxicity is a major player in the secondary injury cascades after SCI [93] the reduction of excitation by ketones may play a significant role in their protective effects after SCI. In addition, neuroprotection by ketones may be related to the upregulation of HIF1α[18]a known regulator of several neuroprotective factors, such as brain-derived neurotrophic factor (BDNF), vascular endothelial growth factor (VEGF), and stromal cell-derived factor -1α (SDF1α). It has been reported that HIF1α expression increases in the injured spinal cord [94], however, we could not detect any differences between SD and KD treated rats in any of the prominent candidate targets downstream of HIF1. While we cannot rule out that our dissection technique might have diluted possible effects; these effects, if existing, are likely small or highly localized since with the same dissection technique we found a robust increase in MCT-1 – that we also confirmed on the mRNA level (data not shown). Very recently, βhydroxybutyric acid, at concentrations that do occur under KD, was shown to inhibit class I Histone Deacetylases (HDAC1 and HDAC2), leading to increased acetylation at the Foxo3a and Mt2 (metallothionein) promoters of genes known to protect against oxidative stress [95]. In addition, this study found induced levels of MnSOD and catalase also known to protect against oxidative stress. 

In summary, we show that short-term post-injury KD treatment provides neuroprotection, and conveys into long-term functional benefits following SCI in rats. Interestingly, KD preserved tissue following the primary impact but this was abolished in the presence of the MCT inhibitor α-cyano-4-hydroxycinnamic acid (4-CIN), suggesting that transport of monocarboxylic acids such as ketone bodies played a role. Although 4-CIN is thought of as a specific inhibitor of MCTs, it is important to note that it also inhibits the mitochondrial pyruvate transporter, as well as the anion exchanger AE1. More research will be necessary to clarify the precise role of MCT1 in spinal cord injury and KD. 

We suggest that KD could be readily translated into the clinical setting of SCI since this dietary regimen is already a well-established therapy for epilepsy, and KD formulas are already available for enteral feeding of human patients (Ketocal; Nutricia, Gaithersburg, MD). Our results call for a reconsideration of standard clinical practices, which have traditionally promoted high carbohydrate nutritional content in “standard enteric formulations” for acute SCI patients [96-99]. We would encourage opening further dialogue on this important aspect of patient care in acute SCI, given the limited interventions currently available for this devastating injury. As dietary treatments like the ketogenic diet most likely evoke a wide array of complex metabolic changes, we do not exclude the possibility that KD may also act through other actions including antioxidant, energy metabolism, programmed cell death and anti-inflammatory effects. As we understand the underlying mechanisms better, it will be possible to develop more palatable dietary approaches or alternative strategies that produce similar or even improved therapeutic effects. As we have to feed the SCI patient, changing a diet to a more protective one can be implemented anywhere in the world at low cost and without major regulatory hurdles.

## References

[1] Plunet WT, Streijger F, Lam CK, Lee JH, Liu J et al. (2008) Dietary restriction started after spinal cord injury improves functional recovery. Exp Neurol 213: 28-35. doi:10.1016/j.expneurol.2008.04.011. PubMed: 18585708. 18585708

[2] Plunet WT, Lam CK, Lee JH, Liu J, Tetzlaff W (2010) Prophylactic dietary restriction may promote functional recovery and increase lifespan after spinal cord injury. Ann N Y Acad Sci 1198 Suppl 1: E1-11. doi:10.1111/j.1749-6632.2010.05564.x. PubMed: 20590533. 20590533

[3] Jeong MA, Plunet W, Streijger F, Lee JH, Plemel JR et al. (2011) Intermittent fasting improves functional recovery after rat thoracic contusion spinal cord injury. J Neurotrauma 28: 479-492. doi:10.1089/neu.2010.1609. PubMed: 21219083. 21219083 PMC3119327

[4] Yudkoff M, Daikhin Y, Melø TM, Nissim I, Sonnewald U (2007) The ketogenic diet and brain metabolism of amino acids: relationship to the anticonvulsant effect. Annu Rev Nutr 27: 415-430. doi:10.1146/annurev.nutr.27.061406.093722. PubMed: 17444813. 17444813 PMC4237068

[5] Guzmán M, Blázquez C (2004) Ketone body synthesis in the brain: possible neuroprotective effects. Prostaglandins Leukot Essent Fatty Acids 70: 287-292. doi:10.1016/j.plefa.2003.05.001. PubMed: 14769487. 14769487

[6] Elwood JC, Marco A, Van Bruggen JT (1960) Lipid metabolism in the diabetic rat. IV. Metabolism of acetate, acetoacetate, butyrate, and mevalonate in vitro. J Biol Chem 235: 573-577. PubMed: 13820133. 13820133

[7] Ito T, Quastel JH (1970) Acetoacetate metabolism in infant and adult rat brain in vitro. Biochem J 116: 641-655. PubMed: 5435493. 5435493 10.1042/bj1160641 PMC1185409

[8] Hawkins RA, Williamson DH, Krebs HA (1971) Ketone-body utilization by adult and suckling rat brain in vivo. Biochem J 122: 13-18. PubMed: 5124783. 5124783 10.1042/bj1220013 PMC1176682

[9] Leino RL, Gerhart DZ, Duelli R, Enerson BE, Drewes LR (2001) Diet-induced ketosis increases monocarboxylate transporter (MCT1) levels in rat brain. Neurochem Int 38: 519-527. doi:10.1016/S0197-0186(00)00102-9. PubMed: 11248400. 11248400

[10] Melø TM, Nehlig A, Sonnewald U (2006) Neuronal-glial interactions in rats fed a ketogenic diet. Neurochem Int 48: 498-507. doi:10.1016/j.neuint.2005.12.037. PubMed: 16542760. 16542760

[11] Pan JW, de Graaf RA, Petersen KF, Shulman GI, Hetherington HP et al. (2002) [2,4-13 C2 ]-beta-Hydroxybutyrate metabolism in human brain. J Cereb Blood Flow Metab 22: 890-898. PubMed: 12142574. 12142574 10.1097/00004647-200207000-00014 PMC2995543

[12] Morris AA (2005) Cerebral ketone body metabolism. J Inherit Metab Dis 28: 109-121. doi:10.1007/s10545-005-5518-0. PubMed: 15877199. 15877199

[13] Kim do Y, Rho JM (2008) The ketogenic diet and epilepsy. Curr Opin Clin Nutr Metab Care 11: 113-120. doi:10.1097/MCO.0b013e3282f44c06. PubMed: 18301085. 18301085

[14] Vanitallie TB, Nonas C, Di Rocco A, Boyar K, Hyams K et al. (2005) Treatment of Parkinson disease with diet-induced hyperketonemia: a feasibility study. Neurology 64: 728-730. doi:10.1212/01.WNL.0000152046.11390.45. PubMed: 15728303. 15728303

[15] Van der Auwera I, Wera S, Van Leuven F, Henderson ST (2005) A ketogenic diet reduces amyloid beta 40 and 42 in a mouse model of Alzheimer's disease. Nutr Metab (Lond) 2: 28. doi:10.1186/1743-7075-2-28. 16229744 PMC1282589

[16] Tai KK, Truong DD (2007) Ketogenic diet prevents seizure and reduces myoclonic jerks in rats with cardiac arrest-induced cerebral hypoxia. Neurosci Lett 425: 34-38. doi:10.1016/j.neulet.2007.08.007. PubMed: 17825488. 17825488

[17] Tai KK, Nguyen N, Pham L, Truong DD (2008) Ketogenic diet prevents cardiac arrest-induced cerebral ischemic neurodegeneration. J Neural Transm 115: 1011-1017. doi:10.1007/s00702-008-0050-7. PubMed: 18478178. 18478178

[18] Puchowicz MA, Zechel JL, Valerio J, Emancipator DS, Xu K et al. (2008) Neuroprotection in diet-induced ketotic rat brain after focal ischemia. J Cereb Blood Flow Metab 28: 1907-1916. doi:10.1038/jcbfm.2008.79. PubMed: 18648382. 18648382 PMC3621146

[19] Prins ML, Fujima LS, Hovda DA (2005) Age-dependent reduction of cortical contusion volume by ketones after traumatic brain injury. J Neurosci Res 82: 413-420. doi:10.1002/jnr.20633. PubMed: 16180224. 16180224

[20] Lee JH, Streijger F, Tigchelaar S, Maloon M, Liu J et al. (2012) A contusive model of unilateral cervical spinal cord injury using the infinite horizon impactor. J Vis Exp. 10.3791/3313 PMC3479980 22871686

[21] Lee JH, Tigchelaar S, Liu J, Stammers AM, Streijger F et al. (2010) Lack of neuroprotective effects of simvastatin and minocycline in a model of cervical spinal cord injury. Exp Neurol 225: 219-230. doi:10.1016/j.expneurol.2010.06.018. PubMed: 20599974. 20599974

[22] Whishaw IQ, Gorny B, Tran-Nguyen LT, Castañeda E, Miklyaeva EI et al. (1994) Making two movements at once: impairments of movement, posture, and their integration underlie the adult skilled reaching deficit of neonatally dopamine-depleted rats. Behav Brain Res 61: 65-77. doi:10.1016/0166-4328(94)90009-4. PubMed: 8031497. 8031497

[23] Liu Y, Kim D, Himes BT, Chow SY, Schallert T et al. (1999) Transplants of fibroblasts genetically modified to express BDNF promote regeneration of adult rat rubrospinal axons and recovery of forelimb function. J Neurosci 19: 4370-4387. PubMed: 10341240. 10341240 10.1523/JNEUROSCI.19-11-04370.1999 PMC6782629

[24] Schallert T, Fleming SM, Leasure JL, Tillerson JL, Bland ST (2000) CNS plasticity and assessment of forelimb sensorimotor outcome in unilateral rat models of stroke, cortical ablation, parkinsonism and spinal cord injury. Neuropharmacology 39: 777-787. doi:10.1016/S0028-3908(00)00005-8. PubMed: 10699444. 10699444

[25] Gensel JC, Tovar CA, Hamers FP, Deibert RJ, Beattie MS et al. (2006) Behavioral and histological characterization of unilateral cervical spinal cord contusion injury in rats. J Neurotrauma 23: 36-54. doi:10.1089/neu.2006.23.36. PubMed: 16430371. 16430371

[26] Montoya CP, Campbell-Hope LJ, Pemberton KD, Dunnett SB (1991) The "staircase test": a measure of independent forelimb reaching and grasping abilities in rats. J Neurosci Methods 36: 219-228. doi:10.1016/0165-0270(91)90048-5. PubMed: 2062117. 2062117

[27] Nikkhah G, Rosenthal C, Hedrich HJ, Samii M (1998) Differences in acquisition and full performance in skilled forelimb use as measured by the 'staircase test' in five rat strains. Behav Brain Res 92: 85-95. doi:10.1016/S0166-4328(97)00128-9. PubMed: 9588688. 9588688

[28] Kloth V, Klein A, Loettrich D, Nikkhah G (2006) Colour-coded pellets increase the sensitivity of the staircase test to differentiate skilled forelimb performances of control and 6-hydroxydopamine lesioned rats. Brain. Res Bull 70: 68-80. doi:10.1016/j.brainresbull.2006.04.006. 16750485

[29] Metz GA, Whishaw IQ (2000) Skilled reaching an action pattern: stability in rat (Rattus norvegicus) grasping movements as a function of changing food pellet size. Behav Brain Res 116: 111-122. doi:10.1016/S0166-4328(00)00245-X. PubMed: 11080542. 11080542

[30] Girgis J, Merrett D, Kirkland S, Metz GA, Verge V et al. (2007) Reaching training in rats with spinal cord injury promotes plasticity and task specific recovery. Brain 130: 2993-3003. doi:10.1093/brain/awm245. PubMed: 17928316. 17928316

[31] Rosen GD, Harry JD (1990) Brain volume estimation from serial section measurements: a comparison of methodologies. J Neurosci Methods 35: 115-124. doi:10.1016/0165-0270(90)90101-K. PubMed: 2283883. 2283883

[32] Puchowicz MA, Xu K, Sun X, Ivy A, Emancipator D et al. (2007) Diet-induced ketosis increases capillary density without altered blood flow in rat brain. Am J Physiol Endocrinol Metab 292: E1607-E1615. doi:10.1152/ajpendo.00512.2006. PubMed: 17284577. 17284577

[33] Streijger F, Beernink TM, Lee JH, Bhatnagar T, Park S et al. (2013) Characterization of a cervical spinal cord hemicontusion injury in mice using the Infinite Horizon Impactor. J Neurotrauma, 30: 869–83. PubMed: 23360150 . 23360150 10.1089/neu.2012.2405

[34] Anderson KD (2004) Targeting recovery: priorities of the spinal cord-injured population. J Neurotrauma 21: 1371-1383. doi:10.1089/neu.2004.21.1371. PubMed: 15672628. 15672628

[35] Sacrey LA, Alaverdashvili M, Whishaw IQ (2009) Similar hand shaping in reaching-for-food (skilled reaching) in rats and humans provides evidence of homology in release, collection, and manipulation movements. Behav Brain Res 204: 153-161. doi:10.1016/j.bbr.2009.05.035. PubMed: 19520119. 19520119

[36] Thio LL, Erbayat-Altay E, Rensing N, Yamada KA (2006) Leptin contributes to slower weight gain in juvenile rodents on a ketogenic diet. Pediatr Res 60: 413-417. doi:10.1203/01.pdr.0000238244.54610.27. PubMed: 16940251. 16940251

[37] Shanley LJ, Irving AJ, Harvey J (2001) Leptin enhances NMDA receptor function and modulates hippocampal synaptic plasticity. J Neurosci 21: RC186: RC186 PubMed: 11734601. 10.1523/JNEUROSCI.21-24-j0001.2001 PMC6763052 11734601

[38] Harvey J, Solovyova N, Irving A (2006) Leptin and its role in hippocampal synaptic plasticity. Prog Lipid Res 45: 369-378. doi:10.1016/j.plipres.2006.03.001. PubMed: 16678906. 16678906 PMC1762032

[39] Irving AJ, Wallace L, Durakoglugil D, Harvey J (2006) Leptin enhances NR2B-mediated N-methyl-D-aspartate responses via a mitogen-activated protein kinase-dependent process in cerebellar granule cells. Neuroscience 138: 1137-1148. doi:10.1016/j.neuroscience.2005.11.042. PubMed: 16413128. 16413128 PMC1613257

[40] Oomura Y, Hori N, Shiraishi T, Fukunaga K, Takeda H et al. (2006) Leptin facilitates learning and memory performance and enhances hippocampal CA1 long-term potentiation and CaMK II phosphorylation in rats. Peptides 27: 2738-2749. doi:10.1016/j.peptides.2006.07.001. PubMed: 16914228. 16914228

[41] Valerio A, Ghisi V, Dossena M, Tonello C, Giordano A et al. (2006) Leptin increases axonal growth cone size in developing mouse cortical neurons by convergent signals inactivating glycogen synthase kinase-3beta. J Biol Chem 281: 12950-12958. doi:10.1074/jbc.M508691200. PubMed: 16522636. 16522636

[42] O'Malley D, MacDonald N, Mizielinska S, Connolly CN, Irving AJ et al. (2007) Leptin promotes rapid dynamic changes in hippocampal dendritic morphology. Mol Cell Neurosci 35: 559-572. doi:10.1016/j.mcn.2007.05.001. PubMed: 17618127. 17618127 PMC1995039

[43] Dasgupta B, Milbrandt J (2007) Resveratrol stimulates AMP kinase activity in neurons. Proc Natl Acad Sci U S A 104: 7217-7222. doi:10.1073/pnas.0610068104. PubMed: 17438283. 17438283 PMC1855377

[44] Kleman AM, Yuan JY, Aja S, Ronnett GV, Landree LE (2008) Physiological glucose is critical for optimized neuronal viability and AMPK responsiveness in vitro. J Neurosci Methods 167: 292-301. doi:10.1016/j.jneumeth.2007.08.028. PubMed: 17936912. 17936912 PMC2257477

[45] Chen DF, Schneider GE, Martinou JC, Tonegawa S (1997) Bcl-2 promotes regeneration of severed axons in mammalian CNS. Nature 385: 434-439. doi:10.1038/385434a0. PubMed: 9009190. 9009190

[46] Leaver SG, Cui Q, Bernard O, Harvey AR (2006) Cooperative effects of bcl-2 and AAV-mediated expression of CNTF on retinal ganglion cell survival and axonal regeneration in adult transgenic mice. Eur J Neurosci 24: 3323-3332. doi:10.1111/j.1460-9568.2006.05230.x. PubMed: 17229081. 17229081

[47] Jiao J, Huang X, Feit-Leithman RA, Neve RL, Snider W et al. (2005) Bcl-2 enhances Ca(2+) signaling to support the intrinsic regenerative capacity of CNS axons. EMBO J 24: 1068-1078. doi:10.1038/sj.emboj.7600589. PubMed: 15719013. 15719013 PMC554135

[48] Bough KJ, Wetherington J, Hassel B, Pare JF, Gawryluk JW et al. (2006) Mitochondrial biogenesis in the anticonvulsant mechanism of the ketogenic diet. Ann Neurol 60: 223-235. doi:10.1002/ana.20899. PubMed: 16807920. 16807920

[49] Mattson MP, Liu D (2003) Mitochondrial potassium channels and uncoupling proteins in synaptic plasticity and neuronal cell death. Biochem Biophys Res Commun 304: 539-549. doi:10.1016/S0006-291X(03)00627-2. PubMed: 12729589. 12729589

[50] Ben-Shachar D, Laifenfeld D (2004) Mitochondria, synaptic plasticity, and schizophrenia. Int Rev Neurobiol 59: 273-296. doi:10.1016/S0074-7742(04)59011-6. PubMed: 15006492. 15006492

[51] Li Z, Okamoto K-I, Hayashi Y, Sheng M (2004) The importance of dendritic mitochondria in the morphogenesis and plasticity of spines and synapses. Cell 119: 873-887. doi:10.1016/j.cell.2004.11.003. PubMed: 15607982. 15607982

[52] Madri JA (2009) Modeling the neurovascular niche: implications for recovery from CNS injury. J Physiol Pharmacol 60 Suppl 4: 95-104. PubMed: 20083857. 20083857

[53] Vavrek R, Girgis J, Tetzlaff W, Hiebert GW, Fouad K (2006) BDNF promotes connections of corticospinal neurons onto spared descending interneurons in spinal cord injured rats. Brain 129: 1534-1545. doi:10.1093/brain/awl087. PubMed: 16632552. 16632552

[54] Kim HM, Hwang DH, Lee JE, Kim SU, Kim BG (2009) Ex vivo VEGF delivery by neural stem cells enhances proliferation of glial progenitors, angiogenesis, and tissue sparing after spinal cord injury. PLOS ONE 4: e4987. doi:10.1371/journal.pone.0004987. PubMed: 19319198. 19319198 PMC2656622

[55] Tysseling VM, Mithal D, Sahni V, Birch D, Jung H et al. (2011) SDF1 in the dorsal corticospinal tract promotes CXCR4+ cell migration after spinal cord injury. J Neuroinflammation 8: 16. doi:10.1186/1742-2094-8-16. PubMed: 21324162. 21324162 PMC3050722

[56] Jaerve A, Bosse F, Müller HW (2012) SDF-1/CXCL12: its role in spinal cord injury. Int J Biochem Cell Biol 44: 452-456. doi:10.1016/j.biocel.2011.11.023. PubMed: 22172378. 22172378

[57] Pierre K, Pellerin L, Debernardi R, Riederer BM, Magistretti PJ (2000) Cell-specific localization of monocarboxylate transporters, MCT1 and MCT2, in the adult mouse brain revealed by double immunohistochemical labeling and confocal microscopy. Neuroscience 100: 617-627. doi:10.1016/S0306-4522(00)00294-3. PubMed: 11098125. 11098125

[58] Kwon BK, Okon E, Hillyer J, Mann C, Baptiste D et al. (2011) A Systematic Review of Non-Invasive Pharmacologic Neuroprotective Treatments for Acute Spinal Cord Injury. J Neurotrauma . 10.1089/neu.2009.1149 PMC3143410 20146558

[59] Kwon BK, Okon EB, Plunet W, Baptiste D, Fouad K et al. (2011) A Systematic Review of Directly Applied Biologic Therapies for Acute Spinal Cord Injury. J Neurotrauma . 10.1089/neu.2009.1150 PMC3143411 20082560

[60] Taub E, Miller NE, Novack TA, Cook EW, Fleming WC, et al (1993) Technique to improve chronic motor deficit after stroke. Arch Phys Med Rehabil 74: 347-354. PubMed: 8466415. 8466415

[61] Taub E (1976) Movement in nonhuman primates deprived of somatosensory feedback. Exerc Sport Sci Rev 4: 335-374. PubMed: 828579. 828579

[62] Tieu K, Perier C, Caspersen C, Teismann P, Wu DC et al. (2003) D-beta-hydroxybutyrate rescues mitochondrial respiration and mitigates features of Parkinson disease. J Clin Invest 112: 892-901. doi:10.1172/JCI200318797. PubMed: 12975474. 12975474 PMC193668

[63] Noh HS, Kim YS, Kim YH, Han JY, Park CH et al. (2006) Ketogenic diet protects the hippocampus from kainic acid toxicity by inhibiting the dissociation of bad from 14-3-3. J Neurosci Res 84: 1829-1836. doi:10.1002/jnr.21057. PubMed: 17058267. 17058267

[64] Mejía-Toiber J, Montiel T, Massieu L (2006) D-beta-hydroxybutyrate prevents glutamate-mediated lipoperoxidation and neuronal damage elicited during glycolysis inhibition in vivo. Neurochem Res 31: 1399-1408. doi:10.1007/s11064-006-9189-5. PubMed: 17115265. 17115265

[65] Massieu L, Haces ML, Montiel T, Hernández-Fonseca K (2003) Acetoacetate protects hippocampal neurons against glutamate-mediated neuronal damage during glycolysis inhibition. Neuroscience 120: 365-378. doi:10.1016/S0306-4522(03)00266-5. PubMed: 12890508. 12890508

[66] Maalouf M, Sullivan PG, Davis L, Kim DY, Rho JM (2007) Ketones inhibit mitochondrial production of reactive oxygen species production following glutamate excitotoxicity by increasing NADH oxidation. Neuroscience 145: 256-264. doi:10.1016/j.neuroscience.2006.11.065. PubMed: 17240074. 17240074 PMC1865572

[67] Kweon GR, Marks JD, Krencik R, Leung EH, Schumacker PT et al. (2004) Distinct mechanisms of neurodegeneration induced by chronic complex I inhibition in dopaminergic and non-dopaminergic cells. J Biol Chem 279: 51783-51792. doi:10.1074/jbc.M407336200. PubMed: 15469939. 15469939

[68] Kashiwaya Y, Takeshima T, Mori N, Nakashima K, Clarke K et al. (2000) D-beta-hydroxybutyrate protects neurons in models of Alzheimer's and Parkinson's disease. Proc Natl Acad Sci U S A 97: 5440-5444. doi:10.1073/pnas.97.10.5440. PubMed: 10805800. 10805800 PMC25847

[69] Imamura K, Takeshima T, Kashiwaya Y, Nakaso K, Nakashima K (2006) D-beta-hydroxybutyrate protects dopaminergic SH-SY5Y cells in a rotenone model of Parkinson's disease. J Neurosci Res 84: 1376-1384. doi:10.1002/jnr.21021. PubMed: 16917840. 16917840

[70] Suzuki M, Kitamura Y, Mori S, Sato K, Dohi S et al. (2002) Beta-hydroxybutyrate, a cerebral function improving agent, protects rat brain against ischemic damage caused by permanent and transient focal cerebral ischemia. Jpn J Pharmacol 89: 36-43. doi:10.1254/jjp.89.36. PubMed: 12083741. 12083741

[71] Davis LM, Pauly JR, Readnower RD, Rho JM, Sullivan PG (2008) Fasting is neuroprotective following traumatic brain injury. J Neurosci Res 86: 1812-1822. doi:10.1002/jnr.21628. PubMed: 18241053. 18241053

[72] Veech RL (2004) The therapeutic implications of ketone bodies: the effects of ketone bodies in pathological conditions: ketosis, ketogenic diet, redox states, insulin resistance, and mitochondrial metabolism. Prostaglandins Leukot Essent Fatty Acids 70: 309-319. doi:10.1016/j.plefa.2003.09.007. PubMed: 14769489. 14769489

[73] Sullivan PG, Rippy NA, Dorenbos K, Concepcion RC, Agarwal AK et al. (2004) The ketogenic diet increases mitochondrial uncoupling protein levels and activity. Ann Neurol 55: 576-580. doi:10.1002/ana.20062. PubMed: 15048898. 15048898

[74] Schültke E, Kendall E, Kamencic H, Ghong Z, Griebel RW et al. (2003) Quercetin promotes functional recovery following acute spinal cord injury. J Neurotrauma 20: 583-591. doi:10.1089/089771503767168500. PubMed: 12906742. 12906742

[75] Hillard VH, Peng H, Zhang Y, Das K, Murali R et al. (2004) Tempol, a nitroxide antioxidant, improves locomotor and histological outcomes after spinal cord contusion in rats. J Neurotrauma 21: 1405-1414. doi:10.1089/neu.2004.21.1405. PubMed: 15672631. 15672631

[76] Genovese T, Mazzon E, Esposito E, Muià C, Di Paola R et al. (2007) Role of endogenous glutathione in the secondary damage in experimental spinal cord injury in mice. Neurosci Lett 423: 41-46. doi:10.1016/j.neulet.2007.05.058. PubMed: 17669594. 17669594

[77] Genovese T, Mazzon E, Menegazzi M, Di Paola R, Muià C et al. (2006) Neuroprotection and enhanced recovery with hypericum perforatum extract after experimental spinal cord injury in mice. Shock 25: 608-617. doi:10.1097/01.shk.0000209560.54328.69. PubMed: 16721269. 16721269

[78] Genovese T, Mazzon E, Muià C, Bramanti P, De Sarro A et al. (2005) Attenuation in the evolution of experimental spinal cord trauma by treatment with melatonin. J Pineal Res 38: 198-208. doi:10.1111/j.1600-079X.2004.00194.x. PubMed: 15725342. 15725342

[79] Duijvestijn AM, van Goor H, Klatter F, Majoor GD, van Bussel E et al. (1992) Antibodies defining rat endothelial cells: RECA-1, a pan-endothelial cell-specific monoclonal antibody. Lab Invest 66: 459-466. PubMed: 1583886. 1583886

[80] Whetstone WD, Hsu JY, Eisenberg M, Werb Z, Noble-Haeusslein LJ (2003) Blood-spinal cord barrier after spinal cord injury: relation to revascularization and wound healing. J Neurosci Res 74: 227-239. doi:10.1002/jnr.10759. PubMed: 14515352. 14515352 PMC2837839

[81] Duelli R, Staudt R, Duembgen L, Kuschinsky W (1999) Increase in glucose transporter densities of Glut3 and decrease of glucose utilization in rat brain after one week of hypoglycemia. Brain Res 831: 254-262. doi:10.1016/S0006-8993(99)01463-8. PubMed: 10412004. 10412004

[82] Duelli R, Kuschinsky W (2001) Brain glucose transporters: relationship to local energy demand. News Physiol Sci 16: 71-76. PubMed: 11390952. 11390952 10.1152/physiologyonline.2001.16.2.71

[83] Noh HS, Lee HP, Kim DW, Kang SS, Cho GJ et al. (2004) A cDNA microarray analysis of gene expression profiles in rat hippocampus following a ketogenic diet. Brain Res. Mol Brain Res 129: 80-87. doi:10.1016/j.molbrainres.2004.06.020. PubMed: 15469884. 15469884

[84] Schurr A, Payne RS, Miller JJ, Tseng MT, Rigor BM (2001) Blockade of lactate transport exacerbates delayed neuronal damage in a rat model of cerebral ischemia. Brain Res 895: 268-272. doi:10.1016/S0006-8993(01)02082-0. PubMed: 11259789. 11259789

[85] Halestrap AP, Price NT (1999) The proton-linked monocarboxylate transporter (MCT) family: structure, function and regulation. Biochem J 343 2: 281-299. doi:10.1042/0264-6021:3430281. PubMed: 10510291. 10510291 PMC1220552

[86] Enerson BE, Drewes LR (2003) Molecular features, regulation, and function of monocarboxylate transporters: implications for drug delivery. J Pharmacol Sci 92: 1531-1544. doi:10.1002/jps.10389. PubMed: 12884241. 12884241

[87] Halestrap AP, Meredith D (2004) The SLC16 gene family-from monocarboxylate transporters (MCTs) to aromatic amino acid transporters and beyond. Pflugers Arch 447: 619-628. doi:10.1007/s00424-003-1067-2. PubMed: 12739169. 12739169

[88] Makuc J, Cappellaro C, Boles E (2004) Co-expression of a mammalian accessory trafficking protein enables functional expression of the rat MCT1 monocarboxylate transporter in Saccharomyces cerevisiae. FEMS Yeast Res 4: 795-801. doi:10.1016/j.femsyr.2004.06.003. PubMed: 15450186. 15450186

[89] Chiry O, Pellerin L, Monnet-Tschudi F, Fishbein WN, Merezhinskaya N et al. (2006) Expression of the monocarboxylate transporter MCT1 in the adult human brain cortex. Brain Res 1070: 65-70. doi:10.1016/j.brainres.2005.11.064. PubMed: 16403470. 16403470

[90] Pellerin L, Pellegri G, Martin JL, Magistretti PJ (1998) Expression of monocarboxylate transporter mRNAs in mouse brain: support for a distinct role of lactate as an energy substrate for the neonatal vs. adult brain. Proc Natl Acad Sci U S A 95: 3990-3995. doi:10.1073/pnas.95.7.3990. PubMed: 9520480. 9520480 PMC19950

[91] Blázquez C, Woods A, de Ceballos ML, Carling D, Guzmán M (1999) The AMP-activated protein kinase is involved in the regulation of ketone body production by astrocytes. J Neurochem 73: 1674-1682. PubMed: 10501215. 10501215 10.1046/j.1471-4159.1999.731674.x

[92] Lutas A, Yellen G (2013) The ketogenic diet: metabolic influences on brain excitability and epilepsy. Trends Neurosci 36: 32-40. doi:10.1016/j.tins.2012.11.005. PubMed: 23228828. 23228828 PMC3534786

[93] Liu D, Xu GY, Pan E, McAdoo DJ (1999) Neurotoxicity of glutamate at the concentration released upon spinal cord injury. Neuroscience 93: 1383-1389. doi:10.1016/S0306-4522(99)00278-X. PubMed: 10501463. 10501463

[94] Xiaowei H, Ninghui Z, Wei X, Yiping T, Linfeng X (2006) The experimental study of hypoxia-inducible factor-1alpha and its target genes in spinal cord injury. Spinal Cord 44: 35-43. doi:10.1038/sj.sc.3101813. PubMed: 16044166. 16044166

[95] Shimazu T, Hirschey MD, Newman J, He W, Shirakawa K et al. (2013) Suppression of oxidative stress by β-hydroxybutyrate, an endogenous histone deacetylase inhibitor. Science 339: 211-214. doi:10.1126/science.1227166. PubMed: 23223453. 23223453 PMC3735349

[96] Rodriguez DJ, Benzel EC (1999) Nutritional support. New York: Churchill Livingstone.

[97] Cruse JM, Lewis RE, Roe DL, Dilioglou S, Blaine MC et al. (2000) Facilitation of immune function, healing of pressure ulcers, and nutritional status in spinal cord injury patients. Exp Mol Pathol 68: 38-54. doi:10.1006/exmp.1999.2292. PubMed: 10640453. 10640453

[98] Riley KO, May AK, Hadley MN (2001) Neurological injury and nutritional support. In: Neurological injury and nutritional support. Philadelphia: Lippincott Wiliams & Wilkins.

[99] CNS CONS (2002) nutritional support after spinal cord injury. Neurosurgery 50: S81-S84. doi:10.1097/00006123-200203001-00015. PubMed: 12431291. 12431291

